# Whole resting cells vs. cell free extracts of *Candida parapsilosis* ATCC 7330 for the synthesis of gold nanoparticles

**DOI:** 10.1186/s13568-016-0268-y

**Published:** 2016-10-07

**Authors:** Saravanan Krishnan, Shoba Narayan, Anju Chadha

**Affiliations:** 1 Laboratory of Bioorganic Chemistry, Department of Biotechnology, Indian Institute of Technology Madras, Chennai, India; 2 Faculty of Allied Health Sciences, Chettinad Academy of Research and Education, Kelambakkam, Chennai, India; 3 National Center for Catalysis Research, Indian Institute of Technology Madras, Chennai, India; 4 Centre for NEMS and Nanophotonics, Indian Institute of Technology Madras, Chennai, India

**Keywords:** *Candida parapsilosis* ATCC 7330, Whole resting cells, Cell free extract, Culture age, Gold nanoparticles, Dispersion stability

## Abstract

The cell free extracts of *Candida parapsilosis* ATCC 7330 are more efficient than the whole resting cells of the yeast in the synthesis of directly usable gold nanoparticles as revealed by this systematic study. Cell free extracts yielded gold nanoparticles of hydrodynamic diameter (50–200 nm). In this study, the total protein concentration influences the nanofabrication and not only the reductase enzymes as originally thought. Powder X-ray diffraction studies confirm the crystalline nature of the gold nanoparticles. Fourier Transform Infra Red spectroscopy and thermal gravimetric analysis suggests that the biosynthesized gold nanoparticles are capped by peptides/proteins. Dispersion experiments indicate a stable dispersion of gold nanoparticles in pH 12 solutions which is also confirmed by electron microscopic analysis and validated using a surface plasmon resonance assay. The effectiveness of the dispersed nanoparticles for the reduction of 4-nitrophenol using sodium borohydride as a reductant further confirms the formation of functional gold nanoparticles. It is also reported that gold nanoparticles with mean particle diameter of 27 nm are biosynthesized inside the whole cell by transmission electron microscopy analysis. With optimized reaction conditions, maximum gold bioaccumulation with the 24 h culture age of the yeast with cellular uptake of ~10^10^ gold atoms at the single cell level is achieved but it is not easy to extract the gold nanoparticles from the whole resting cells.

**Electronic supplementary material:**

The online version of this article (doi:10.1186/s13568-016-0268-y) contains supplementary material, which is available to authorized users.

## Introduction

Microorganisms play a key role in the removal of toxic heavy metals and metalloids from the polluted environment by biosorption, bioaccumulation, biotransformation and biomineralization (Dixit et al. 2015; Gadd 2010; Reith et al. 2007). There is also an emphasis on using microbes for the synthesis of nanoparticles as compared to chemical methods since the microbe mediated methods are environmentally benign and sustainable (Sharma and Mudhoo 2010; Singh 2015; Virkutyte and Varma 2013). Among the microbes, fungi are well known for heavy metal binding, uptake and accumulation (Dighton and White 2005). Fungal species are efficient biological systems for the synthesis of metal nanoparticles (Boroumand Moghaddam et al. 2015). Among the noble metal nanoparticles, gold nanoparticles are widely used for diverse applications in diagnosis, therapy and catalysis (Eustis and El-Sayed 2006). Compared to bacteria, synthesis of gold nanoparticles using fungal systems has advantages such as the ease of handling/scale up, presence of redox enzymes and capping agents for enhanced productivity (Yadav et al. 2015). The initial report on fungus mediated preparation of gold nanoparticles used the *Verticillium* sp. where the nanoparticles are formed intracellularly (Mukherjee et al. 2001) but the isolation of nanoparticles from the cells is still quite a challenge.

*Pichia jadinii* (Gericke and Pinches 2006b) produces spherical gold nanoparticles intracellularly whereas *Y. lipolytica* (Agnihotri et al. 2009) biosynthesized nanoparticles remain associated with the cell wall. Besides spherical gold nanoparticles, yeast species are also reported to produce nanoparticles of varying size and morphology e.g. triangular and hexagonal gold nanostructures are produced with extracts of *V. volvacea* (Philip 2009). Using yeast species, formation of cadmium sulfide (Dameron et al. 1989), lead sulfide (Seshadri et al. 2011), titanium dioxide (Jha et al. 2009a) and antimony oxide (Jha et al. 2009b) nanoparticles are also reported.

Microbial production i.e., whole resting/fermenting cells, cell free extracts, isolated proteins and culture supernatants can be used for the production of nanoparticles. Whole intact cells which are harvested from the culture media are commonly referred to as resting cells and cell free extract is the crude fraction obtained after lysing the resting cells. *Candida parapsilosis* ATCC 7330 is reportedly an established biocatalyst for various organic biotransformations to produce optically pure secondary alcohols and amines (Mahajabeen and Chadha 2013; Venkataraman and Chadha 2015). It is also observed that the biocatalytic activity of this yeast is found to vary with the culture age used for the biotransformation (Kaliaperumal 2011). There are not too many detailed reports on the correlation between the microbial culture age and biogenesis of gold nanoparticles. The bacteria *Thermus scotoductus* SA-01 shows that the maximum removal of Au in the form of nanoparticles occurs during the late exponential phase (Erasmus et al. 2014). In a separate study, the synthesis of silver nanoparticles using the cell free filtrates obtained from 4 to 7 days culture of *Penicillium nalgiovense* AJ12 shows the importance of culture age (Wang and Chen 2009). Even though these reports have specified the importance of microbial culture age, the other parameters which affect the metal bioaccumulation are not dealt with. This study shows the optimization of parameters such as culture age, biomass concentration and incubation time for the biosynthesis of gold nanoparticles using the intact whole cells of *Candida parapsilosis* ATCC 7330 and the utility of the cell free extract prepared from the resting cells for the biosynthesis of gold nanoparticles. In addition, experiments to improve the monodispersity of particles formed by the yeast cell free extract is presented.

## Materials and methods

### General

Gold (III) chloride (99 % pure) and Bradford reagent were procured from Sigma Aldrich. Yeast malt broth media constituents (peptone, yeast extract, malt extract; dextrose) and phenyl methane sulfonyl fluoride (PMSF) were purchased from Hi Media laboratories. Buffering reagents such as potassium dihydrogen phosphate and dipotassium hydrogen phosphate were purchased from Merck. Enzyme cofactors such as Nicotinamide adenine dinucleotide (NADH) and Nicotinamide adenine dinucleotide phosphate (NAD(P)H) were procured from Sisco Research Laboratory chemicals. All set of experiments was performed using ultra pure MilliQ H_2_O (resistivity: 18.2 MΩ cm). All the reaction flasks were washed with aqua regia (3: 1 v/v of Conc. hydrochloric acid and nitric acid) before use.

### Microorganism growth conditions and growth behavior in liquid medium

*Candida parapsilosis* ATCC 7330 strain was procured from ATCC, Manassas, VA 20108, USA and grown in yeast malt broth (YMB) media consisting of 5 g L^−1^ peptone, 3 g L^−1^ yeast extract, 3 g L^−1^ malt extract and 10 g L^−1^ dextrose. Yeast malt agar slant was prepared at monthly intervals to maintain stock culture under sterile conditions. For preculture, about two loops (4 mm diameter) was inoculated in sterilised 50 mL media and incubated in an orbital shaker at 200 rpm at 25 °C. For the main culture, about 4 % v/v of preculture was inoculated into sterilised 50 mL YMB broth medium and incubated for respective growth periods according to the aforementioned conditions. The yeast culture and preservation conditions were followed as per established procedures (Kaliaperumal et al. 2010). The growth behavior of *Candida parapsilosis* ATCC 7330 in liquid medium was studied by monitoring the optical density (OD_600_) and dry cell weight as a function of time. Samples were diluted for optical density measurements so as to keep the OD values lesser than 1.0.

### Biosynthesis of gold nanoparticles using resting cells

Different culture ages of the yeast (24, 36 and 48 h) of 50 g L^−1^ concentration were used for the biosynthesis of gold nanoparticles. To the cell suspension prepared with phosphate buffer (pH 7.0, 20 mM), gold (III) chloride was added to make an overall concentration of 1 mM. The reaction flask was maintained at 37 °C, 200 rpm for 72 h. The amount of Au removal from the aqueous solution was determined for different culture ages. After 72 h reaction, the yeast cell suspension was removed from the reaction mixture and the remaining supernatant was used to estimate the amount of gold left over. Through mass balance calculations, the amount of gold loaded into the yeast cells was determined.

In order to determine the optimum cellular concentration, the biomass concentration was varied for the fixed metallic precursor and the amount of gold uptake and cellular morphology was examined. Identical reactions were carried out with biomass concentration ranging from 25 to 150 g L^−1^ for a fixed gold(III) ion concentration (1 mM) and the reaction flasks are incubated for 72 h (200 rpm, 37 °C). Samples were analyzed for gold uptake. For the cases with higher gold uptake, transmission electron microscopic analysis was carried out.

Kinetics of gold accumulation was studied using whole resting cells (20 mL, 50 g L^−1^) for gold (III) chloride (1 mM). At few intervals, the samples were centrifuged to collect the supernatant and the residual gold was measured. Control experiment involves the same concentration of gold precursor added to the buffer and incubated under identical conditions.

The amount of gold atoms present in a single *Candida parapsilosis* cell was determined by equating the cell count to the amount of gold removal by the cells. Number of yeast cells in the 50 g L^−1^ cell suspension was determined using haemocytometer. The average number of cells (per mL) was found to be 1.8 (±0.13) × 10^9^ cells. To the reaction flask, varying number of cells was added, to which gold (III) chloride was added to the flasks and kept for incubation at 37 °C, 200 rpm for 72 h. The amount of gold chloride added was 3.51 × 10^20^ Au atoms. The amount of gold uptake by the cells is determined.

### Biosynthesis of gold nanoparticles using the cell free extract

Yeast cultures grown up to 24 h was harvested to remove the growth media using centrifugation (12,800×*g*, 5 min, 4 °C) and washed twice with MilliQ water, to remove the media components. Phosphate buffer (pH 7, 20 mM) was used to prepare the cell suspension of 50 g L^−1^ concentration. To the cell suspension prepared with 24 h culture, 1 mM phenyl methyl sulfonyl fluoride was added as a protease inhibitor. Cell suspension was then subjected to ultrasonication (Vibra-cell ultrasonicator) under these conditions (1 s pulse on/off, 35 % amplitude, 4 °C, 10 min). After ultrasonication, the suspension was subjected to centrifugation (12,800×*g*, 15 min and 4 °C) and the supernatant was collected. The supernatant fraction was the cell free extract used for the biosynthesis of gold nanoparticles.

Cell free extract prepared from resting cells harvested at 24 h was used for the synthesis of gold nanoparticles. To 20 mL of the prepared extract, gold (III) chloride was added to make an overall concentration of 1 mM. The reaction mixture was then kept for incubation at 37 °C, 200 rpm for 72 h.

The concentration of proteins in the cell free extract was estimated using Bradford assay (Bradford 1976). Alcohol dehydrogenase activity (ADH) (Peters et al. 1993) in the extract was assayed using acetophenone as a model substrate. The consumption of NADH during the reaction was monitored spectrophotometrically (V-530 UV/vis Spectrophotometer) at 340 nm and 25 °C using molar extinction coefficient of 6.22 mL μmol^−1^ cm^−1^. Similarly, glutathione reductase activity (GR) (Romero-Puertas et al. 2006) in the extract was determined using oxidized glutathione as a substrate and NADPH as a cofactor. One unit (U) of ADH or GR activity is defined as the amount of the enzyme that catalyses the oxidation of 1 μmol of NAD(P)H per minute respectively under the conditions specified.

The cell free extract prepared from the resting cells of *Candida parapsilosis* ATCC 7330 (fraction I) was used as a reference. The reaction mixture after 72 h was subjected to centrifugation (18,500×*g*, 60 min, 4 °C). The supernatant fraction containing the unbound proteins (UBP) was collected. The pellet had the gold nanoparticles. The protein concentration in the cell free extracts i.e. fraction I and the unbound protein fraction was estimated using Bradford assay. Both these fractions were further concentrated by lyophilization; these samples of identical protein amounts (50 µg) were denatured and run through sodium dodecyl sulfate–polyacrylamide gel electrophoresis (12 %). The gel was stained initially using Coomassie staining followed by destaining. In order to improve the resolution, the gel was subjected to quick silver staining.

### Effect of protein concentration on nanofabrication

Preliminary experiments on biosynthesis of gold nanoparticles were carried out by varying the protein concentration (220–2000 µg mL^−1^) for the fixed gold (III) chloride (1 mM). Further, identical reactions were carried out with varying protein concentration (220, 330 and 530 µg mL^−1^), to each of which different concentrations of gold (III) chloride (0.5, 0.75 and 1.0 mM) were added. Experiments were carried out with 20 mL of the prepared cell free extract put in 150 mL conical flask. Reaction flasks were kept for incubation under the standard reaction conditions (37 °C, 200 rpm and 72 h).

### Purification of the biosynthesized gold nanoparticles

Gold nanoparticles were purified from the reaction mixture using centrifugation (18,500×*g*, 60 min, 25 °C), solid nanoparticles were dispersed in MilliQ water and the process was repeated again to obtain gold nanoparticles free from unreacted gold ions.

### Effect of pH on the dispersed gold nanoparticles

About 1.5 mL of the gold nanoparticles was subjected to centrifugation (18,500×*g*, 60 min, 4 °C) and the solid nanoparticles were dispersed in different pH solutions (2, 4, 6, 8, 10, 12). In this study, pH was adjusted in MilliQ water to make different pH solutions (using either hydrochloric acid or sodium hydroxide). Samples were analyzed after the dispersion of gold nanoparticles in respective pH solutions. In addition, long term stability of the better dispersed gold nanoparticles was monitored after 20 months storage at 4 °C.

### Catalytic studies

In this model study, pH 12 dispersed gold nanoparticles was used to study the catalytic reduction of 4-nitrophenol (4-NP) using sodium borohydride (NaBH_4_) as a electron donor at ambient conditions. The progress of the reduction of 4-NP was monitored spectrophotometrically as a decrease in the absorbance of the substrate 4-NP at 400 nm. In the cuvette assay, the final concentrations of the 4-nitrophenol and NaBH_4_ were made 10^−4^ and 10^−2^ M respectively. Typically, this second order reaction was made pseudo first order by keeping NaBH_4_ concentration 100 fold excess in relative to 4-nitrophenol. About 10 μL (3.3 × 10^−9^ mol) of the gold nanoparticles was added and the final volume was adjusted to 1 mL using MilliQ water. Identical reactions without the addition of gold nanoparticles were performed as a control experiment. Time course of the catalysis was studied through monitoring the absorbance at 400 nm.

### Characterization methods

Characteristic surface plasmon resonance peak of the gold nanoparticles was monitored using JASCO V-530 UV/vis Spectrophotometer. The amount of gold removed by the biomass from the aqueous solution was determined using induced coupled plasma-optical emission spectrometry (Perkin Elmer Optima 5300 DV). The residual concentration of gold in the reaction supernatants was determined by recording the optical emission at λ/nm (267.595), intrinsic to the gold element. Prior to the analysis, instrument was calibrated with known concentration of gold solution. Hydrodynamic diameter, polydispersity index and zeta potential of the gold nanoparticles were determined using Zetasizer 3000 HSA (Malvern Instruments, UK). All these characterization experiments were done in triplicate samples and the results were provided with respective mean value and standard deviation.

Transmission electron microscopy was performed using Philips CM12 Transmission electron microscope to examine the size and morphology of the gold nanoparticles. For better understanding, Image J analysis (Image J 1.46r) was performed to determine the primary particle size from the electron micrographs. The phase identity and crystalline nature of the gold nanoparticles were determined with Bruker Discover D8 diffractometer using Cu (Kα) radiation (λ/Å = 1.5406) at room temperature in the 2θ range from 30° to 100°. The surface chemistry of the lyophilised gold nanoparticles was analysed using FT-IR Eco-ATR Bruker ALPHA spectrometer in the range 600–4000 *ν*_*max*_/cm^−1^ with a resolution of 4 cm^−1^. Thermal decomposition of the lyophilized gold nanoparticles was analyzed using thermal gravimetry (NETZSCH STA 449F3) instrument in nitrogen atmosphere at the rate of heating of 5 °C per minute with Al_2_O_3_ as a reference sample.

## Results

### Whole resting cells mediated Au NPs biosynthesis

#### Effect of culture age

**Fig. 1:**
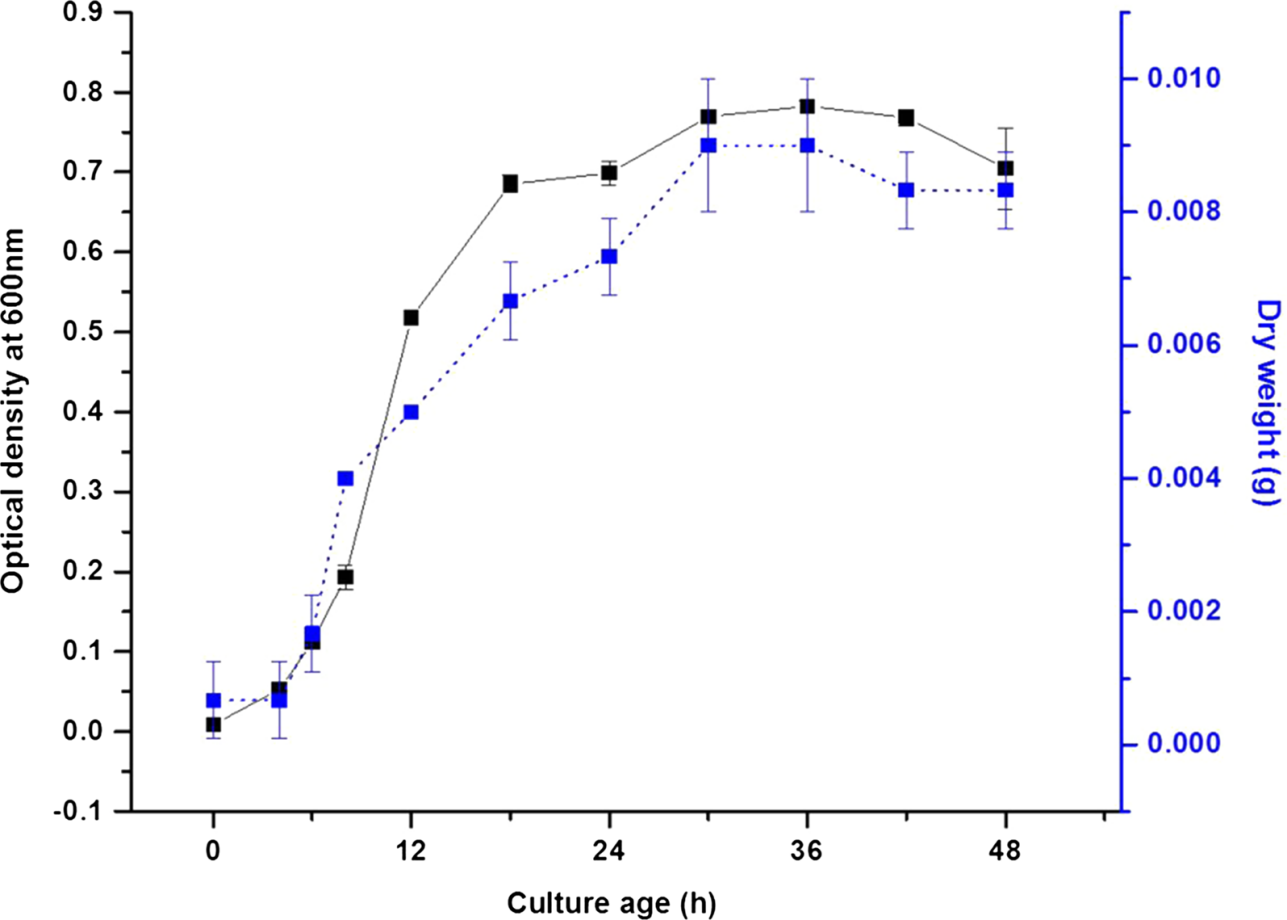
Growth curve of *Candida parapsilosis* ATCC 7330 cultured in a shake flask using Yeast Malt Broth media

**Fig. 2:**
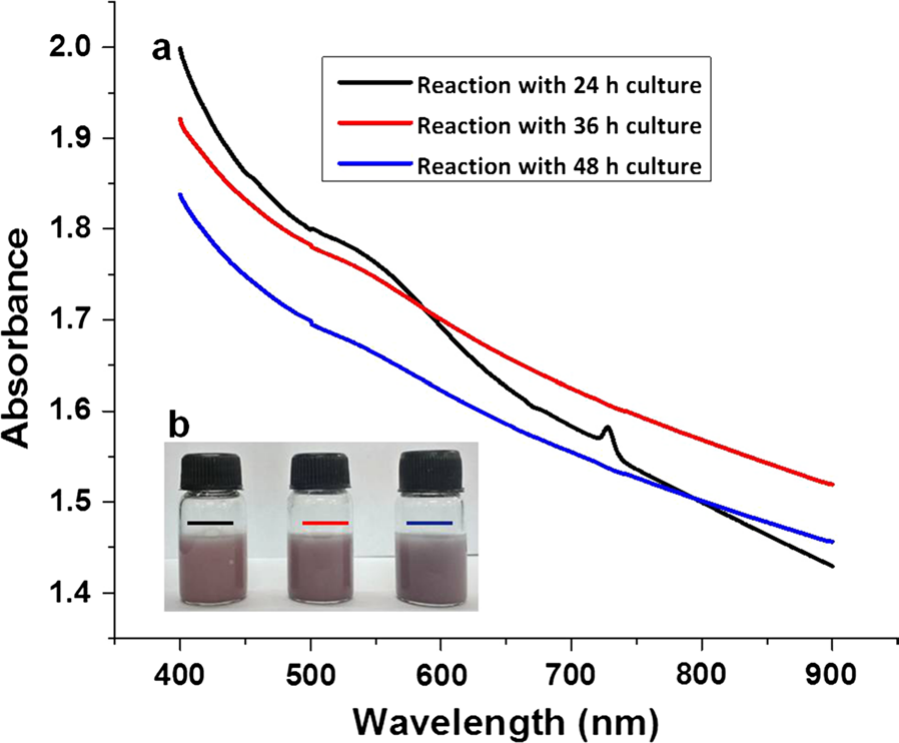
**a** UV/vis spectrum analysis of the gold nanoparticles biosynthesized using whole cells (24, 36 and 48 h culture cells) of *Candida parapsilosis* ATCC 7330, **b** visual representation of the reaction

The growth of this yeast in yeast malt broth media (YMB) shows the lag phase (0–6 h), exponential/log phase (6–30 h), stationary phase (30–42 h) and decline phase (42 h and above) (Fig. 1). Fermenting cells which contain AuCl_3_ did not show any color associated with gold nanoparticles formation. Yeast biomass harvested at different culture ages (24, 36 and 48 h) were incubated with 1 mM AuCl_3_. UV/vis spectrum analysis of biomass showed the formation of gold nanoparticles as seen spectrophotometrically at ~520–580 nm (surface plasmon resonance, SPR) (Fig. 2a). The initial color of the biomass (pale white) gradually changed and a pinkish coloration was observed with all culture ages (Fig. 2b). Visible inspection of the reaction biomass showed that the pink color is retained with the biomass. The reaction supernatants (free from cell biomass) did not show the spectrophotometric response (~520–580 nm) of gold nanoparticles formation which indicates the lack of extracellular formation of nanoparticles. From quantitative analysis of the reaction supernatants, it was found that the gold removal from the aqueous solution was 77 % with 24 h resting biomass and there was no major difference in the gold removal from the solution with increasing culture ages i.e., 36 h (76 %) and 48 h (77 %).

**Fig. 3:**
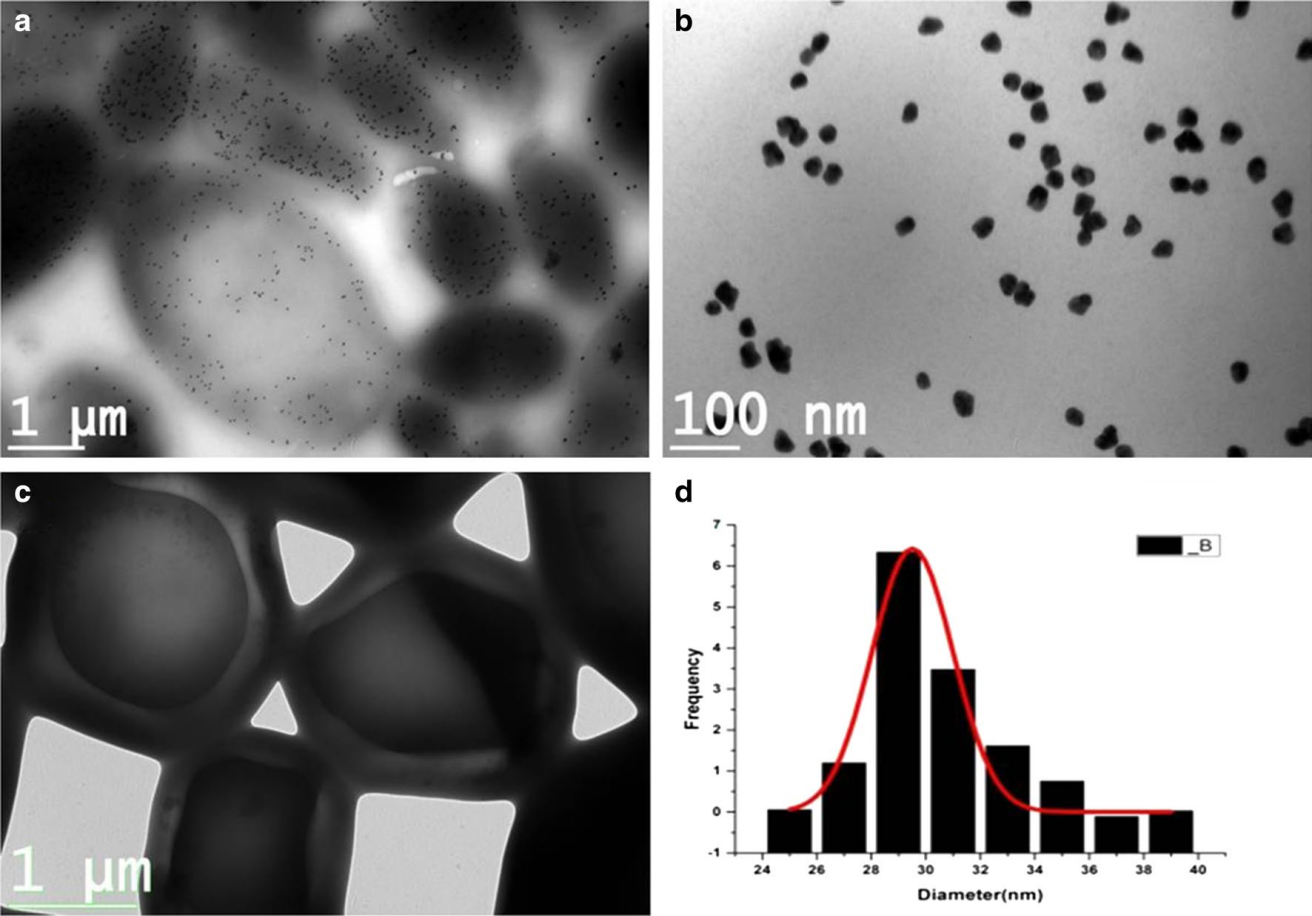
**a** Transmission electron micrographs of the whole cell mounts after the reaction (*Scale bar* 1 μm, 3000×), **b** enlarged micrographs (*Scale bar* 100 nm, 28,000×), **c** control cells (*Scale bar* 1 μm, 5000×) and **d** frequency based size distribution of gold nanoparticles

Transmission electron microscopy analysis of the whole cell mounts shows that the gold nanoparticles were formed using the resting cells (24 h culture) of *Candida parapsilosis* ATCC 7330 (Fig. 3a) Gold nanoparticles biosynthesized using the resting cells of 24 h culture were spherical in shape. The enlarged micrographs show the uniform distribution of nanoparticles with average size of about 27 nm (Fig. 3b, d) on the whole cells. Energy dispersive analysis of the reaction with resting biomass shows the optical absorption at 2 keV which is a characteristic of the metallic gold which was not observed for the control cells (Fig. 3c; Additional file 1: Figure S1a, b). Experiments showed that the heat killed cells did not produce any discrete nanoparticles (Additional file 1: Figure S2).

### Influence of biomass concentration

**Fig. 4:**
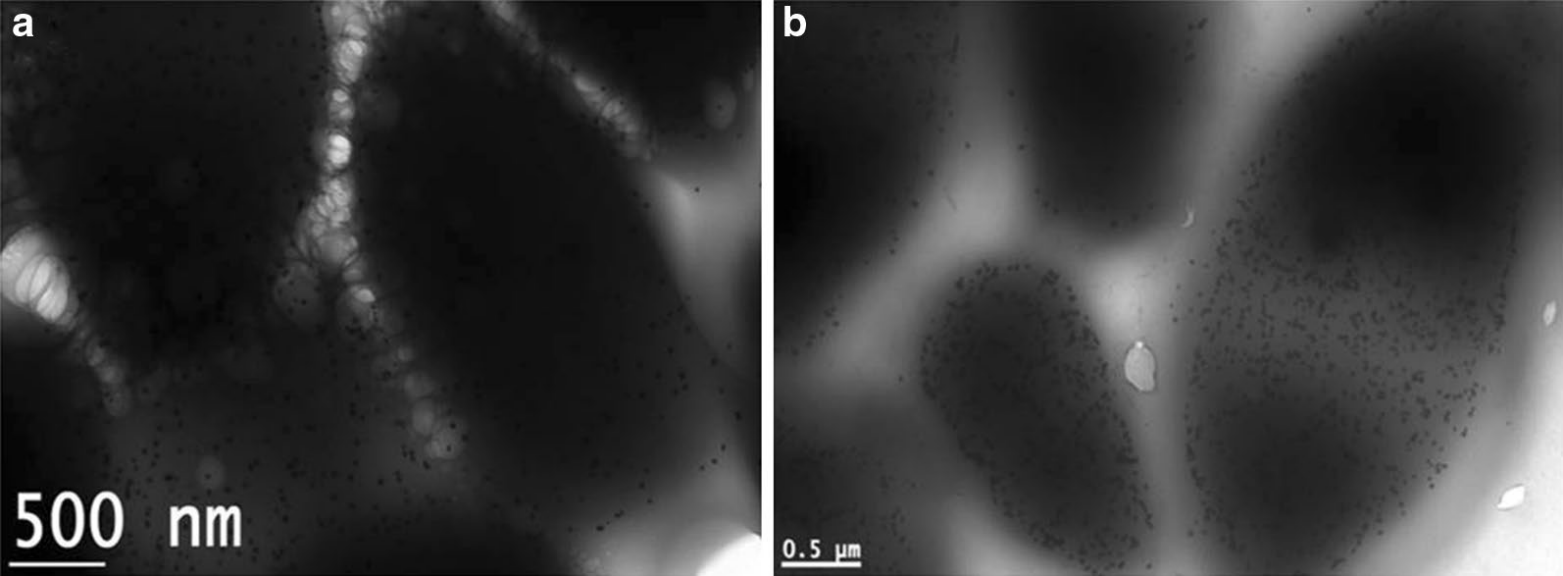
Transmission electron microscopic analysis of the gold nanoparticles biosynthesized using **a** 25 g L^−1^ and **b** 50 g L^−1^ of the biomass concentration against 1 mM AuCl_3_ (*Scale bar* 500 nm)

In the present study, different concentrations of the *Candida parapsilosis* ATCC 7330 were used for a fixed gold (III) concentration (1 mM). About 79 and 76 % of gold (III) was removed (Additional file 1: Figure S3) from the aqueous solution for the biomass concentrations of 25 and 50 g L^−1^ respectively and the cell morphology remain almost intact as visualized from electron micrographs (Fig. 4a, b). For the biomass concentrations of 75 and 150 g L^−1^, the gold uptake decreased to 63 and 52 % respectively.

### Kinetics of gold accumulation vs. biomineralization of gold nanoparticles

**Fig. 5:**
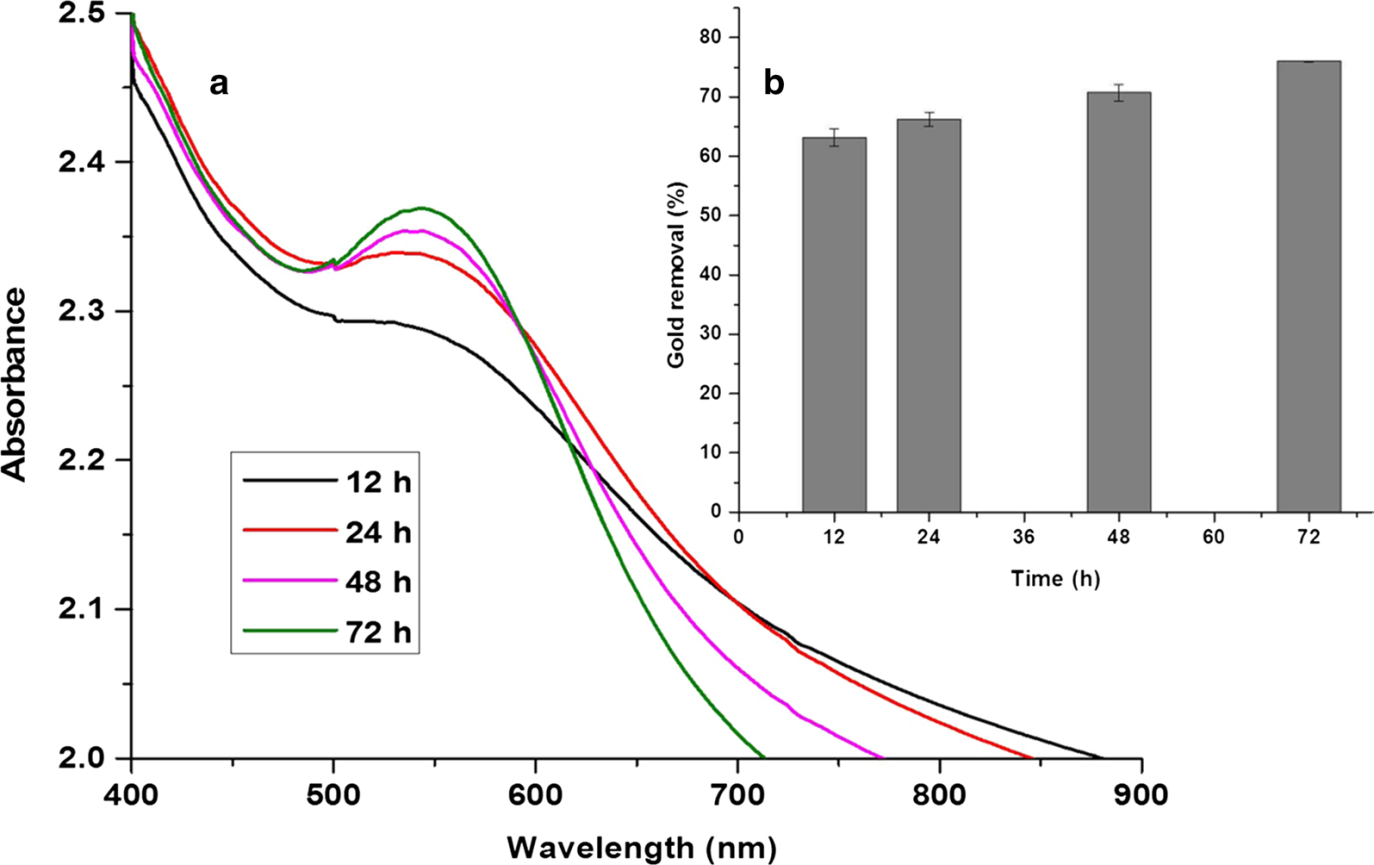
Kinetics of intracellular formation of gold nanoparticles using resting cells of *Candida parapsilosis* ATCC 7330 **a** UV/vis spectrum analysis and **b** Gold accumulation with respect to time

Gold accumulation kinetics was studied for the biomass concentration of 50 g L^−1^ against 1 mM AuCl_3_. UV/vis spectrum analysis showed that the biomineralization of gold nanoparticles was increasing with time and more noticeably from 24 to 72 h (Fig. 5a). About 77 % of the gold was bioaccumulated at the end of 72 h (Fig. 5b).

Optical density (OD) measurements were initially calibrated against the cell number (Additional file 1: Figure S4) such that 1 OD corresponds to 5.4 × 10^7^ cells (0.366 g DCW/L). Assuming that all cells were identical and the cell number was constant during the course of reaction, the maximum number of gold atoms accumulated as nanoparticles within a single cell was found to be in the order of 10^10^ atoms (Additional file 1: Table S1). Efforts to extract the gold nanoparticles formed within the resting biomass using ultrasonication (Additional file 1: Table S2) gave poor yields of stable Au NPs.

### Cell free extract mediated Au NPs biosynthesis

**Fig. 6:**
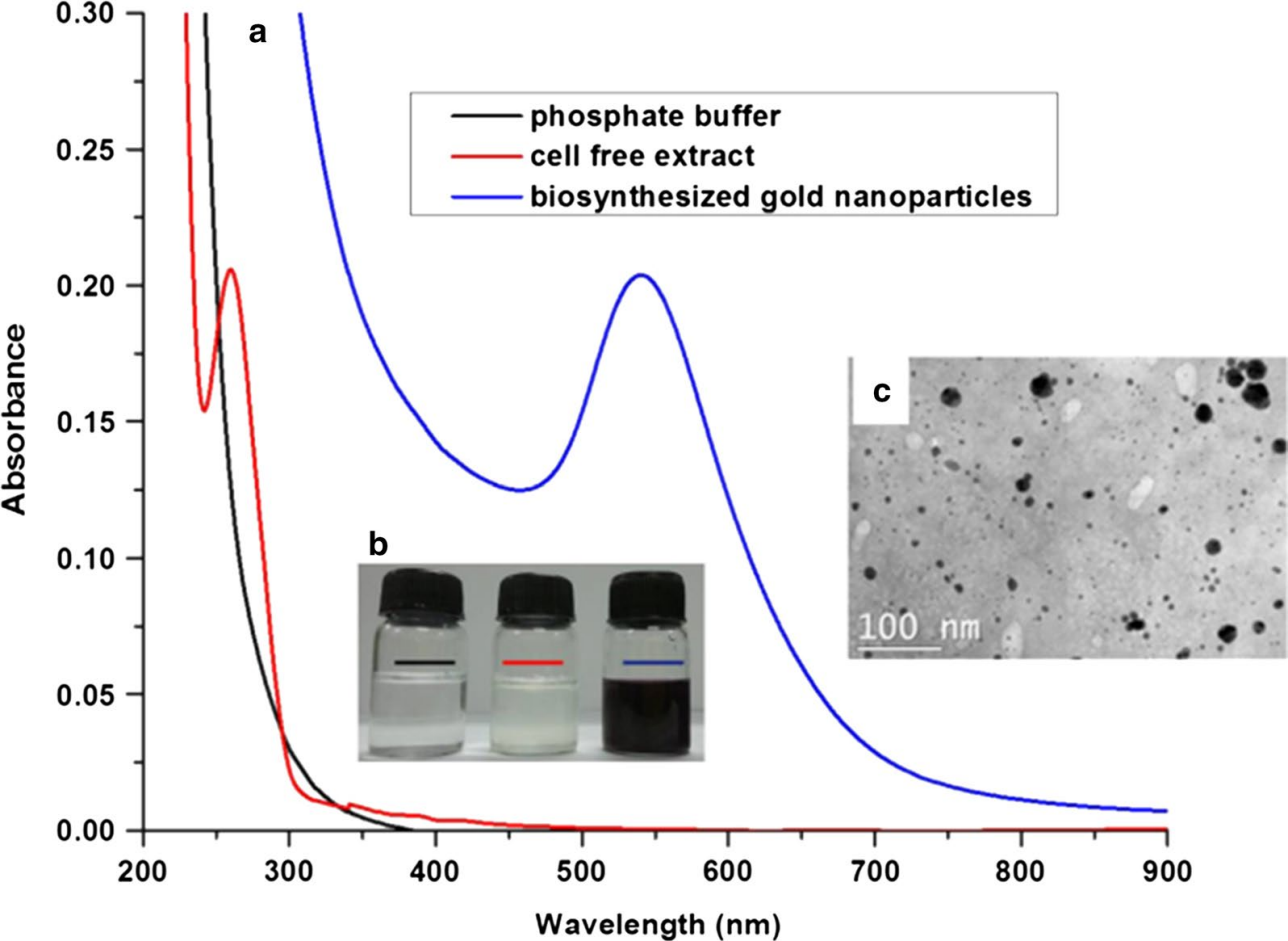
**a** UV/vis spectrum of the biosynthesis of gold nanoparticles using the cell free extract of *Candida parapsilosis* ATCC 7300, **b** Visual inspection and **c** Electron micrographs of the gold nanoparticles formed using cell free extract (*Scale bar* 100 nm, 35 K magnification)

Under identical experimental conditions, the cell free extract of 24 h culture prepared from 50 g L^−1^ cell suspension was used instead of resting cells. Using UV/vis spectrophotometer, SPR peak (535 nm) corresponding to the formation of gold nanoparticles was observed which was also consistent with the visual inspection (Fig. 6a, b). Gold nanoparticles biosynthesized using the cell free extract were spherical as visualized from microscopic images (Fig. 6c). Using Image J software, the primary particles size from the electron micrographs was found to be 52 nm (considering mainly the larger size fraction) which was well in agreement with the hydrodynamic diameter (D_h_) estimation (D_h_, 59 nm) and polydispersity index (PDI) of 0.4. Powder X-ray diffraction study depicts the formation of the Au NPs with low surface energy facets such as (111) and (100) (Additional file 1: Figure S5) which represents their face cubic crystalline nature (ICDD card no. 00-04-0784).

Zeta potential of the biosynthesized gold nanoparticles was found to be −24 mV which indicates colloidal stability. Freezed dried pellet of gold nanoparticles showed FT-IR signals at 3271, 2909, 1641, 1537, 1389, 1234 and 1039 cm^−1^ (Additional file 1: Figure S6). Thermal gravimetric analysis revealed that there was about 10 % weight loss due to the mass change at temperatures in the range of 350–400 °C (Additional file 1: Figure S7). From SDS-PAGE analysis, it was observed that few bands (around 45–60 kDa) diminished in the UBP fraction as compared to fraction 1 (Additional file 1: Figure S8). Protein concentration in fraction 1 was found to be 350 µg mL^−1^ whereas UBP fraction contains 230 µg mL^−1^. There was a difference in protein concentration of about 120 µg/mL (34 % of the total proteins) between fraction I and UBP fraction. Relative quantitation of the gel (50 µg of proteins loaded in each well) using densitometry analysis (Image Lab 3.0 software) showed that the intensity of protein bands (labeled as B1, B2 and B3) in unbound proteins fraction were diminished (Additional file 1: Table S3).

### Effect of protein concentration on biogenesis of gold nanoparticles

**Fig. 7:**
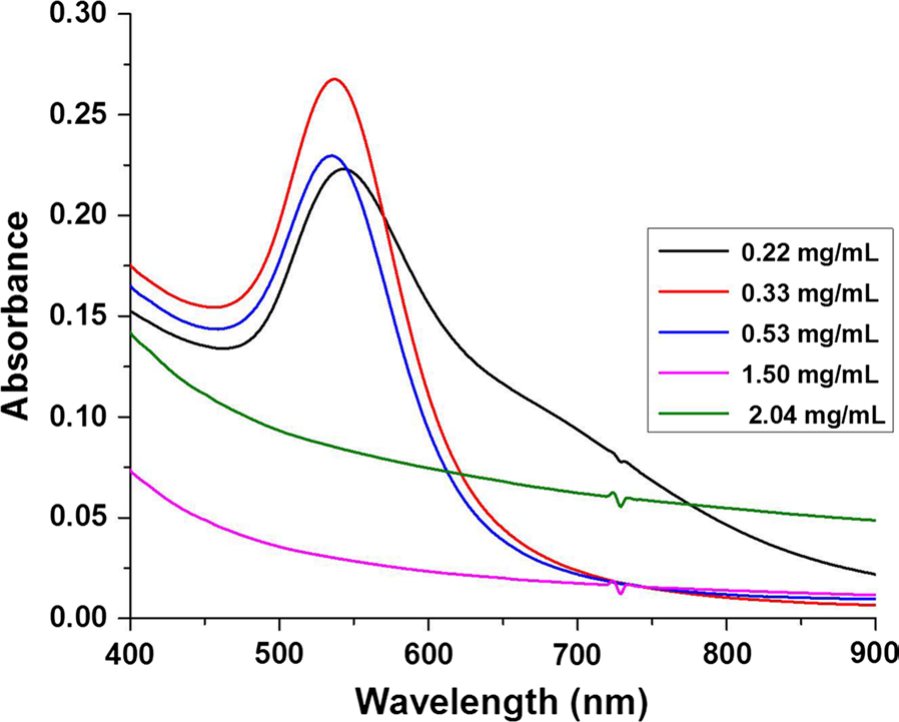
UV/vis spectrum of the gold nanoparticles biosynthesized using varying protein concentrations for a fixed gold precursor

**Fig. 8:**
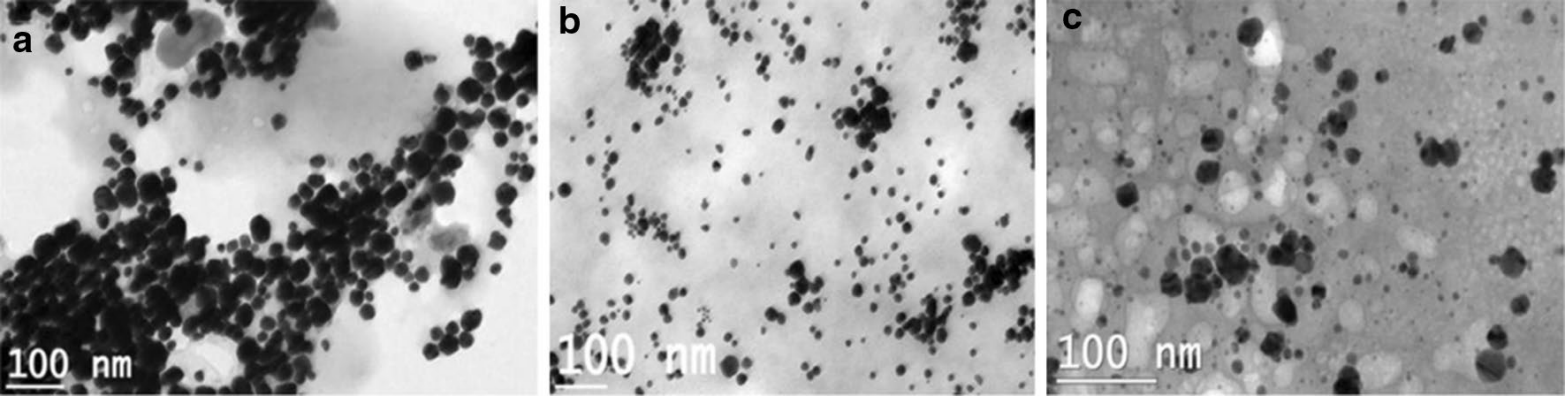
Electron micrographs of the gold nanoparticles biosynthesized through varying protein concentrations (**a** 0.22;** b** 0.33 and** c** 0.53 mg mL^−1^)for a fixed gold precursor (*Scale bar* 100 nm)

The cell free extract contains a variety of enzymes/proteins and other organic molecules. The protein concentration in the cell free extract prepared from 50 g L^−1^ cell suspension was 537 µg mL^−1^. The effect of protein concentration (220–2000 µg mL^−1^) on the biosynthesis of gold nanoparticles was investigated spectrophotometrically. Characteristic surface plasmon resonance (~520–580 nm) corresponding to the formation of nanogolds (Fig. 7) was seen with lower concentrations of the protein (220, 330 and 530 µg mL^−1^). Reactions employing higher protein concentration (1500 and 2000 µg mL^−1^) did not show the SPR bands which indicates that the gold nanoparticles were not formed (Fig. 7). Transmission electron microscopy studies confirmed that the spherical gold nanoparticles formed with 330 and 530 µg mL^−1^ protein fraction were better dispersed (Fig. 8b, c). However, aggregates of gold nanoparticles were observed with 220 µg mL^−1^ protein (Fig. 8a) which was also supported by the secondary broad SPR observed in the higher wavelength (Fig. 7).

Furthermore, each of the protein concentrations i.e., 220, 330 and 530 µg mL^−1^ was titrated against different concentrations of the gold precursor (0.5, 0.75 and 1 mM AuCl_3_) and the effect on the hydrodynamic diameter, polydispersity index and zeta potential of the gold nanoparticles formed was examined. Spherical Au NPs with particle D_h_ ranging from 50 to 222 nm were formed (Table 1). Notably, zeta potential of the Au NPs synthesized for all the reactions showed considerable stability i.e., −27 to −20 mV (Table 1).

**Table 1:** Influence of protein concentration (220–530 µg mL^−1^) in the cell free extract of *Candida parapsilosis* ATCC 7330 against different concentrations of gold precursor (0.5–1 mM) towards the biogenesis of gold nanoparticles

Entry	Protein concentration (μg mL^−1^)	Gold (III) chloride (mM)	Gold nanoparticles			
			SPR band (nm)	Size		Zeta potential (mV)
				D_h_ (nm)	PDI	
1	220 ± 18	0.5	536	57.3 ± 5.8	0.445	−21.4 ± 1.5
2	220 ± 18	0.75	539	109.9 ± 3.	0.308	−27.3 ± 3.2
3	220 ± 18	1.0	543	222 ± 7.7	0.489	−27.8 ± 0.5
4	330 ± 27	0.5	534	56.7 ± 1.1	0.454	−20.8 ± 1.5
5	330 ± 27	0.75	538	71.6 ± 0.6	0.402	−26.2 ± 1.1
6	330 ± 27	1.0	537	50.1 ± 0.6	0.422	−25.5 ± 1.5
7^a^	530 ± 32	0.5	–	–	–	–
8	530 ± 32	0.75	546	164.1 ± 3.	0.429	−21.7 ± 1.4
9	530 ± 32	1.0	535	59.2 ± 1.1	0.433	−24 ± 0.3

^a^No gold nanoparticles formation

Enzymatic assays showed that the cell free extract was enriched in both the reductase enzymes i.e. alcohol dehydrogenase (ADH, 0.047 U mg^−1^) and glutathione reductase (GR, 0.024 U mg^−1^). Experiments showed that at 0.5 mM gold(III) chloride, and a protein concentration of 537 μg mL^−1^ in the reaction mixture failed to produce Au NPs (entry 7 of Table 1). Assuming the reduction of Au^+3^ to Au^0^ is enzyme mediated; the increase in protein concentration should enhance the biosynthesis of Au NPs. On the contrary, increasing protein concentration for a fixed concentration of gold (III) chloride did not improve the biosynthesis of Au NPs. An appropriate control experiment in which heat killed extract was used instead of cell free extract also gave reduced nanogolds (data not shown). Gold nanoparticles produced using cell free extract were polydisperse in nature (Table 1).

### Dispersion studies and colloidal stability

Au NPs were purified and the solid nanoparticles were dispersed in MilliQ water. The dispersed Au NPs in water showed increased D_h_ (76 ± 5 nm) with wavelength maxima of 551 nm as compared to as-prepared nanoparticles (D_h_ of 59 nm, λ_max_ of 535 nm). The polydispersity index of the water dispersed Au NPs (0.47) did not change much with that of the as-prepared Au NPs with PDI (0.42). Zeta potential of water dispersed Au NPs was about −36 ± 3 mV.

**Fig. 9:**
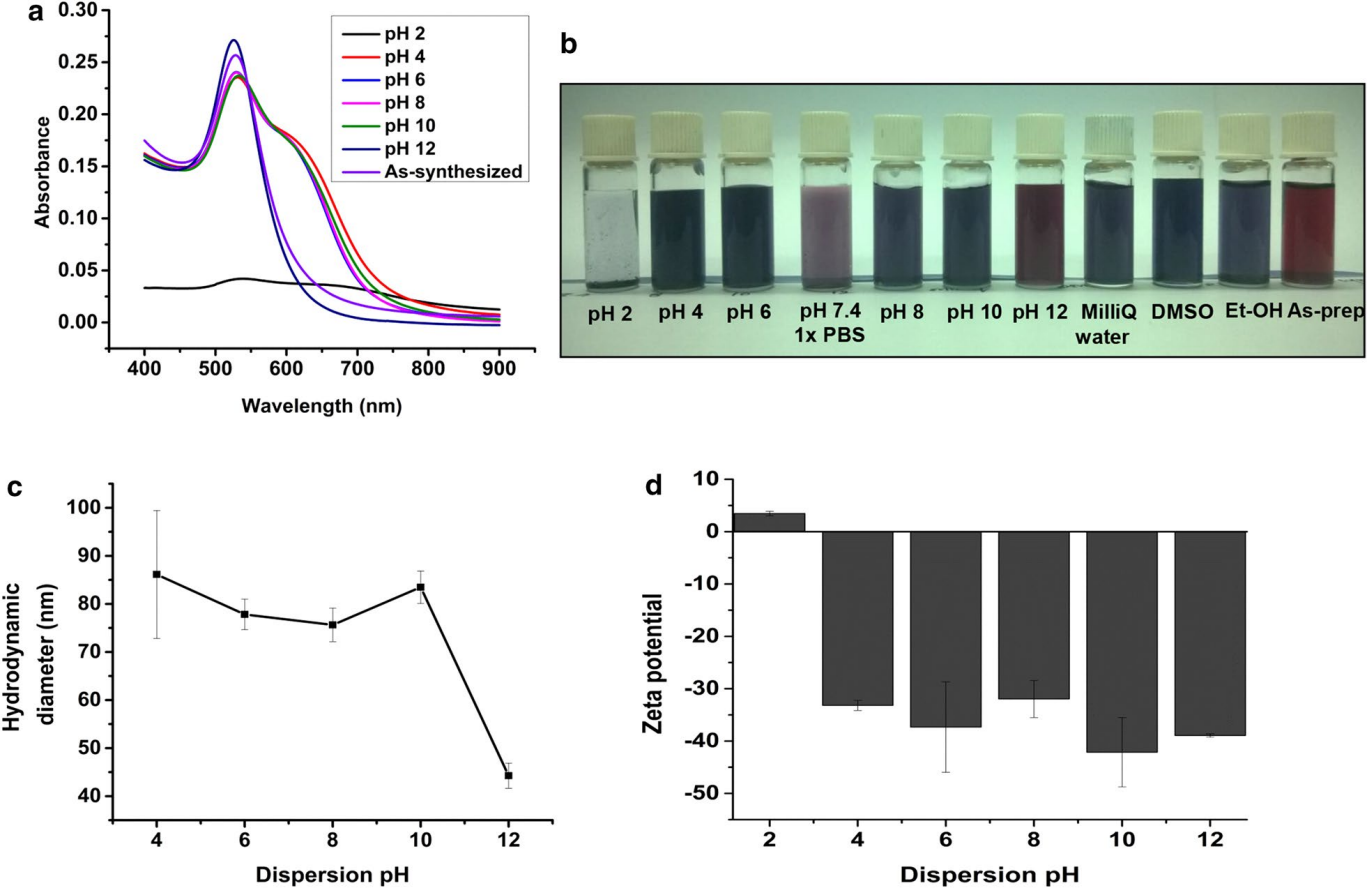
Effect of dispersion of the biosynthesized gold nanoparticles in different pH solutions **a** surface plasmon resonance analysis **b** visual inspection of the dispersed samples **c** dispersion effect on particle hydrodynamic diameter **d** dispersion effect on zeta potential

The dispersion behavior of the biosynthesized Au NPs in various pH solutions was monitored using UV/vis spectrophotometer (Fig. 9a). The difference in the SPR absorbance of the dispersed Au NPs was attributed to their dispersion stability which was also consistent with the visual appearances of the samples (Fig. 9b). The colloidal stability of the pH 12 dispersed Au NPs was evidenced by the 10 nm blue shift from *λ*_*max*_ of 535 nm (as-prepared) to 525 nm.

**Fig. 10:**
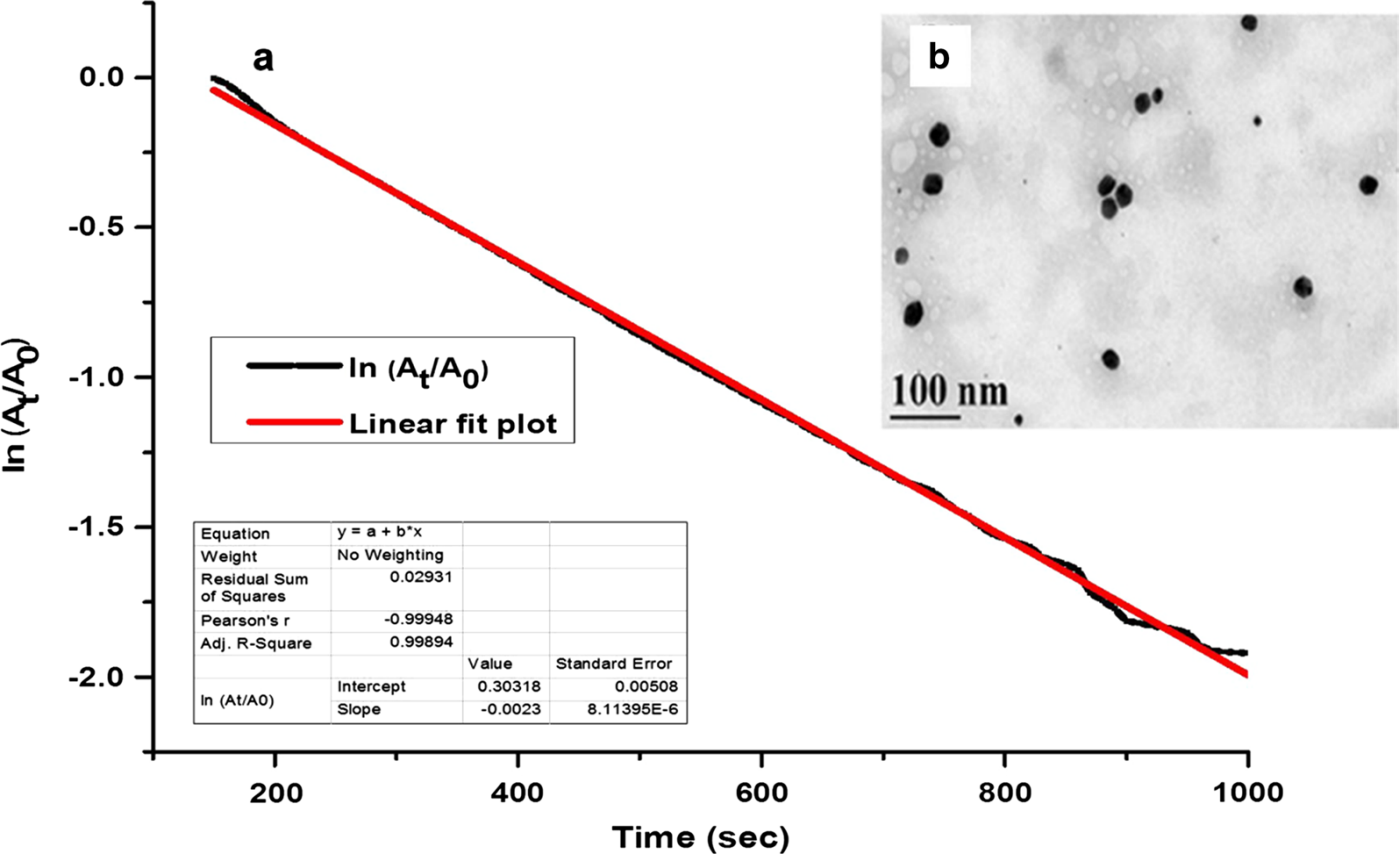
**a** A* plot* showing the linear relationship between ln (A_t_/A_0_) and time for the dispersed (pH 12) gold nanoparticles mediated catalytic reduction of 4-nitrophenol using sodium borohydride as a reductant and **b** transmission electron micrographs of the biosynthesized Au NPs dispersed in pH 12 solution (*Scale bar* 100 nm, 35 k magnification)

Particle size, particle size distribution and zeta potential of the dispersed nanoparticles were measured as a function of pH of the dispersion medium (Fig. 9c, d; Additional file 1: Figure S9). At pH 12, hydrodynamic diameter of the Au NPs was 44.25 with polydispersity index of 0.2 and an increased negative zeta potential of −38.93 mV. Stabilization aspects seems to depend on pH as seen at pH 12 dispersion decreases the polydispersity index of the biosynthesized gold nanoparticles. Electron micrographs confirm the better monodispersity (PDI of 0.2 by dynamic light scattering analysis) of Au NPs in pH 12 solution (Fig. 10b; Additional file 1: Figure S9). Dispersed Au NPs in pH 12 solutions showed a decrease in the diameter and the average size was found to be 32 nm (calculated using Image J software). Storage stability of the pH 12 dispersed gold nanoparticles after 20 months showed a decrease in primary particle size from 32 to 29 nm (Additional file 1: Figure S10) with PDI of 0.2 and zeta potential of −35 mV.

For the pH values of 4–10, the D_h_ is found between 75 and 86 nm and zeta values range from −31 to −42 mV. Dispersion of Au NPs at pH 2 shows gold aggregates with D_h_ of 8530 nm and zeta potential of 3.5 mV. The dispersal of Au NPs in phosphate buffered saline (pH 7.4) yielded nanoparticles with D_h_ of 150 ± 42 nm (Additional file 1: Figure S11) and zeta potential of −20.8 ± 1.5 mV. In addition to aqueous solutions, dispersion of biosynthesized Au NPs in organic polar solvents such as dimethylsulfoxide (DMSO) and ethanol was studied. Dispersion in DMSO yielded Au NPs with D_h_ of 135 ± 6 nm (Additional file 1: Figure S11) and zeta potential of −27 ± 1 mV. In case of ethanol, more gold aggregates were formed with particle D_h_ of 646 nm (Additional file 1: Figure S11) and zeta potential of −23 mV.

The pH 12 system showed abs_520/600_ value of 4.40 i.e. more positive stability as compared to the other pH solutions and as-prepared Au NPs (3.25) (Additional file 1: Figure S12). In the present study, the biosynthesized Au NPs dispersed in pH 12 solution was used as a nanocatalyst for the sodium borohydride mediated reduction of 4-nitrophenol which is a well established model reaction (Panigrahi et al. 2007) for evaluating catalytic activity of the metal nanoparticles. The rapid decrease in the absorbance of 4-nitrophenolate ion was observed with the increase in time as compared to the control experiment (Additional file 1: Figures S13, S14) performed in the absence of gold nanoparticles. A linear plot of ln (A_t_/A_0_) vs. time gave the apparent rate constant (Fig. 10a) (*K*_*app*_) of 2.3 × 10^−3^ s^−1^ (0.138 min^−1^).

## Discussion

Microbial production of nanoparticles involves parameters such as the selection of the suitable biological species and the optimal growth/cultivation conditions necessary for enhanced biosynthesis. When the microbes are challenged with a metallic precursor, mostly aerobic cells use their defense machinery to detoxify the metal which occurs preferably by the reduction of the metal from its native toxic form to the less toxic form (Weast 1984). During this detoxification process, the monomers required for the formation of metal nanoparticles are generated which subsequently undergo nucleation and form nanoparticles. Intracellular accumulation of metals occurs with metabolically active cells (Wang and Chen 2009). More likely, the accumulated gold gets converted to gold nanoparticles within the cell. With resting cells of *Candida parapsilosis* ATCC 7330, gold nanoparticles formed within the cell and the process could be metabolism dependent. A recent study with the fungus *Rhizopus oryzae* shows that gold nanoparticles are biosynthesized on the cell wall and the cytoplasmic region (Das et al. 2012b). Reportedly, biogenesis of gold nanoparticles using *G. candidum* is mediated intracellularly (Mittal et al. 2013). Besides fungal species, intracellular growth of gold nanoparticles are also reported using bacteria (Ivanov et al. 2009) algae (Gangula et al. 2011) and cancerous cell lines (Wang et al. 2013). There is a tremendous interest towards the intracellular synthesis of the metal/alloy nanoparticles using microbes as they are reportedly used as surface enhanced Raman scattering probes (Shamsaie et al. 2007) and heterogeneous catalyst (Deplanche et al. 2012) for chemical reactions.

In this study, the 24 h culture age of *Candida parapsilosis* ATCC 7330 which corresponds to the late exponential phase was found to be optimum for further studies. Culture age (36 and 48 h) of *Candida parapsilosis* ATCC 7330 did not show any increase in the gold removal (76 and 77 %) from the aqueous solution as compared to 24 h culture age. Few reports have correlated the richness of enzyme/proteins present in the exponential phase of the microbes to the maximum number of nanoparticles present in situ. An isolated study (Gericke and Pinches 2006a) showed that the number of gold nanoparticles formed per cell decreases with increase in the culture age of *V. luteoalbum* used. In a separate study, growth phase (exponential phase) of the bacteria is correlated with the maximum amount of gold accumulated within the biomass (Erasmus et al. 2014). With the increase in the biomass concentration, amount of gold (III) loaded into the biomass decreased from 79 % (25 g L^−1^) to 52 % (150 g L^−1^). About 5 % decrease in gold uptake is observed with the dried cells of *Azolla filiculoides* for the increase in biomass concentration from 1 to 9 mg L^−1^ (Antunes et al. 2001). Interferences between the binding sites for microbial biosorption of metals are one of the possible explanations for the decreased metal uptake. Very likely, an increase in biomass concentration decreases the electrostatic interactions between the metal and the cells (Roussos et al. 2013).

The removal of metals from the aqueous solution is strongly influenced by the contact time between the microbe and the metals (Farhan and Khadom 2015). Quantitative kinetics of the gold accumulation indicated that about 77 % Au^+3^ was taken up by the cell after 72 h. Visual monitoring shows that the biomineralization of gold nanoparticles increases with time and the same was in consensus with the SPR intensity. An isolated study shows that the bioaccumulation of radioactive gold (H^198^AuCl_4_) reaches a maximum limit after 10 h incubation with the yeast *Saccharomyces cerevisiae* (Panigrahi et al. 2007). *Candida parapsilosis* ATCC 7330 showed better atom economy (~10^10^) in terms of the gold atoms bioaccumulated within a single yeast cell as compared to AP22 and CCFY-100 strains of the yeast *Saccharomyces cerevisiae* where 10^7^ radioactive gold atoms (^198^Au) are accumulated in the form of nanoparticles of size (15–20 nm) within each cell (Sen et al. 2011).

Apart from resting cells, microbial resources such as crude extracts, cell free filtrates, and purified enzymes/proteins are also used to synthesize metal nanoparticles. Using cell free extracts of *Candida parapsilosis* ATCC 7330, spherical gold nanoparticles with hydrodynamic diameter of 50–220 nm was obtained by varying the reaction stoichiometry between the gold precursor (0.5–1 mM) and the protein concentration (220–530 µg/mL). The biosynthesis of spherical and non-spherical shaped gold nanoparticles in the size of 20–80 nm using the cytosolic extract of *Candida albicans* (Chauhan et al. 2011) is reported. The possible surface chemistry involved in stabilization of gold nanoparticles (lyophilized sample) was studied using FT-IR analysis. A broad band at 3271 cm^−1^ indicated the N–H stretching of the primary amines in the protein molecules (Correa-Llantén et al. 2013). The FT-IR bands noticed at 1641, 1537, 1234 cm^−1^ corresponded to the amide I, amide II and amide III bands of peptide units of polypeptide/proteins respectively (Rajeshkumar et al. 2013; Aryal et al. 2006; Shankar et al. 2003). A band at 1389 cm^−1^ corresponded to the symmetric stretching of the carboxylate (COO^−^) groups (Aryal et al. 2006). A notable band was seen at 1039 cm^−1^ which indicated the C-N stretching vibrations possibly from aliphatic amines of the proteins (Rajeshkumar et al. 2013). This analysis indicated that the peptide/protein molecules could be involved in capping the Au NPs formed. Green synthesis of nanoparticles using microbial extracts are reported to synthesize stable gold nanoparticles where peptide/protein molecules are capping the nanoparticles (Kitching et al. 2014). Thermal decomposition studies of the freeze dried Au NPs samples suggested the presence of capping agents which decomposes around 350–400 °C. A similar finding is reported in a biogenic approach using *C. albicans* (Ahmad et al. 2013), where the protein biomolecules are shown to stabilize the nanoparticles produced. In addition, SDS-PAGE analysis suggest that cellular proteins are involved in the stabilization of the gold nanoparticles biosynthesized using the cell free extract of *Candida parapsilosis* ATCC 7330.

The two main events in gold nanoparticles formation in the yeast, viz. reduction and stabilization (Capping) are recognized but their mechanism is not completely understood (Duran et al. 2011; Faramarzi and Sadighi 2013; Hulkoti and Taranath 2014; Singh et al. 2015). The cell free extract used for the biosynthesis of gold nanoparticles was enriched in alcohol dehydrogenase (ADH) and glutathione reductase (GR) enzymes. It is known that ADH (Drauz et al. 2012) mediates the enzymatic reduction of carbonyl compounds using NAD(P)H whereas GR (Penninckx 2002) catalyzes the formation of reduced glutathione from oxidized glutathione using NADPH. Enzymes such as nitrate reductase (Kalimuthu et al. 2008), hydrogenase (Riddin et al. 2009), sulfate reductase (Ahmad et al. 2002), phenol oxidizing enzymes namely laccase, tyrosinase, Mn-peroxidase (Vetchinkina et al. 2014), alpha-amylase (Manivasagan et al. 2015) and few others are implicated in the bioreduction of Au(III) to Au(0). Vaidyanathan et al. (2010) have shown that optimizing the activity of nitrate reductase enhanced the silver nanoparticles production using *B. licheniformis*. Scott et al. (2008) proposed that the metallic gold nanoparticles formation occurs at the active site of NADPH dependent glutathione reductase. *E. coli* cells bioengineered with bacterial glycerol dehydrogenase are reported for in situ biocatalytic synthesis of gold nanoparticles using NADH as a cofactor (Niide et al. 2011). Reduction of gold precursor was observed even with heat denatured cell free extract which indicate that the process is not entirely due to enzymatic reactions. Thus, multiple reduction/stabilization reactions do occur and produce the reduced nanogolds. This was further supported by the experiment where increasing protein concentration for a fixed metal precursor did not enhance the formation of gold nanoparticles and the same is consistent with earlier reports (Das et al. 2012a; Tan et al. 2010). Thus, direct correlation to reductase activity may not be precise but the total protein concentration likely influences the formation of stable Au NPs with moderate polydispersity.

It is also established that the fungal/yeast species generates metal nanoparticles with broad size distribution (Kitching et al. 2014). The presence of more than one nucleation event during biogenic nanofabrication contributes to polydispersity. The utility of the polydisperse gold nanoparticles is limited due to the overlapping properties of the varying sizes of the nanoparticles produced. Achieving dispersions of gold nanoparticles with narrow size distribution is important. Dispersion of the biosynthesized gold nanoparticles in pH 12 solution improved the monodispersity of the gold nanoparticles. Long term storage of pH 12 dispersed gold nanoparticles for about 20 months at 4 °C show improved dissolution of nanoparticles due to a decrease in the size of the dispersed nanoparticles from 32 to 29 nm (as calculated from electron micrographs) although there was no significant change in the polydispersity index and zeta potential of the nanoparticles. In basic conditions, the deprotonation of the Au-OH groups on the surface of the nanoparticles (Pfeiffer et al. 2014) provides electrostatic repulsive forces improving the dispersion of gold nanoparticles. In the chemical approach, adjusting the reaction to alkaline pH is reported to produce monodisperse gold nanoparticles with controlled particle size distribution (Ji et al. 2007; Kumar et al. 2007; Yang et al. 2007). On the other hand, less positive zeta values (3.5 ± 0.5 mV) of the pH 2 dispersed gold nanoparticles indicate that the thickness of the electrical double layer is drastically reduced (Sennett and Olivier 1965) and therefore as a result, strong aggregation of gold nanoparticles was seen which may be due to the increased Van der Waal’s attractive forces as compared to the stabilizing forces (Nel et al. 2009). Similar observation is reported in the aggregation behavior of biologically synthesized silver nanoparticles (Prathna et al. 2011). For the dispersion of Au NPs in polar solvents such as DMSO and ethanol, the varying agglomeration behavior was observed which could be attributed to the difference in the dielectric constants among the solvents. The results are in accordance with a recent report (Burrows et al. 2014), which states that the decrease in the dielectric constant (DMSO, 48 and ethanol, 24) is expected to decrease the repulsive forces between the nanoparticles and thus results in more aggregation. Colloidal stability of Au NPs is evaluated on the basis of the stability parameter (Ivanov et al. 2009) derived from surface plasmon resonance assay i.e., the ratio of the absorbance measured at 520 nm to that of 600 nm. Surface Plasmon resonance assay confirmed the better dispersion stability of biosynthesized gold nanoparticles in pH 12 solution as compared to other pH solutions. In the presence of sodium borohydride, dispersed gold nanoparticles (in pH 12 solution) was catalytically active as the substrate 4-nitrophenol was found decreasing with time in the spectrophotometric assay and the calculated rate constant was similar to the reported (Zayed and Eisa 2014) values (3.1 × 10^−3^ s^−1^) for Au NPs of 32 nm.

In summary, a one-pot sustainable method employing the use of yeast cells, *Candida parapsilosis* ATCC 7330 as a mild reagent for the biosynthesis of gold nanoparticles was established. Cell free extracts gave better gold nanoparticles of size (50–220 nm) whereas resting cells yielded gold nanoparticles accumulated within the cell. The formation of nanoparticles using the cell free extract depends on total cellular protein concentration and not only enzymatic activity of redox enzymes. This is the first detailed report to address the dispersion behavior of biosynthesized gold nanoparticles in different matrices. Dispersion of the biosynthesized gold nanoparticles in pH 12 solution (32 nm Au NPs) improves the particle monodispersity which is demonstrated via the size dependent catalytic activity for the reduction of 4-nitrophenol.

## Additional file

**Additional file 1.** Additional figures and tables.

## Abbreviations

YMB: yeast malt broth
AuCl_3_: gold (III) chloride
4-NP: 4-nitrophenol
NaBH_4_: sodium borohydride
OD: optical density
Au NPs: gold nanoparticles
SPR: surface plasmon resonance
D_h_: hydrodynamic diameter
PDI: polydispersity index
ADH: alcohol dehydrogenase
GR: glutathione reductase
DMSO: dimethyl sulfoxide
NADH: reduced form of nicotinamide adenine dinucleotide
NAD(P)H: reduced form of nicotinamide adenine dinucleotide phosphate

## References

[CR1] Agnihotri M, Joshi S, Kumar AR, Zinjarde S, Kulkarni S (2009). Biosynthesis of gold nanoparticles by the tropical marine yeast *Yarrowia lipolytica* NCIM 3589. Mater Lett.

[CR2] Ahmad A, Mukherjee P, Mandal D, Senapati S, Khan MI, Kumar R, Sastry M (2002). Enzyme mediated extracellular synthesis of CdS nanoparticles by the fungus, *Fusarium oxysporum*. J Am Chem Soc.

[CR3] Ahmad T, Wani IA, Manzoor N, Ahmed J, Asiri AM (2013). Biosynthesis, structural characterization and antimicrobial activity of gold and silver nanoparticles. Colloids Surf B.

[CR4] Antunes APM, Watkins GM, Duncan JR (2001). Batch studies on the removal of gold(III) from aqueous solution by *Azolla filiculoides*. Biotechnol Lett.

[CR5] Aryal S, Remant BK, Dharmaraj N, Bhattarai N, Kim CH, Kim HY (2006). Spectroscopic identification of S-Au interaction in cysteine capped gold nanoparticles. Spectrochim Acta Mol Biomol Spectrosc.

[CR6] Boroumand Moghaddam A, Namvar F, Moniri M, Md Tahir P, Azizi S, Mohamad R (2015). Nanoparticles biosynthesized by fungi and yeast: a review of their preparation, properties, and medical applications. Molecules.

[CR7] Bradford MM (1976). A rapid and sensitive method for the quantitation of microgram quantities of protein utilizing the principle of protein-dye binding. Anal Biochem.

[CR8] Burrows ND, Kesselman E, Sabyrov K, Stemig A, Talmon Y, Penn RL (2014). Crystalline nanoparticle aggregation in non-aqueous solvents. CrystEngComm.

[CR9] Chauhan A, Zubair S, Tufail S, Sherwani A, Sajid M, Raman SC, Azam A, Owais M (2011). Fungus-mediated biological synthesis of gold nanoparticles: potential in detection of liver cancer. Int J Nanomed.

[CR10] Correa-Llantén DN, Muñoz-Ibacache SA, Castro ME, Muñoz PA, Blamey JM (2013). Gold nanoparticles synthesized by *Geobacillus* sp. strain ID17 a thermophilic bacterium isolated from Deception Island, Antarctica. Microb Cell Fact.

[CR11] Dameron CT, Smith BR, Winge DR (1989). Glutathione-coated cadmium-sulfide crystallites in *Candida glabrata*. J Biol Chem.

[CR12] Das SK, Dickinson C, Lafir F, Brougham DF, Marsili E (2012). Synthesis, characterization and catalytic activity of gold nanoparticles biosynthesized with *Rhizopus oryzae* protein extract. Green Chem.

[CR13] Das SK, Liang J, Schmidt M, Laffir F, Marsili E (2012). Biomineralization mechanism of gold by zygomycete fungi *Rhizopus oryzae*. ACS Nano.

[CR14] Deplanche K, Merroun ML, Casadesus M, Tran DT, Mikheenko IP, Bennett JA, Zhu J, Jones IP, Attard GA, Wood J, Selenska-Pobell S, Macaskie LE (2012). Microbial synthesis of core/shell gold/palladium nanoparticles for applications in green chemistry. J R Soc Interface.

[CR15] Dighton J, White J (2005). The fungal community: its organization and role in the Ecosystem.

[CR16] Dixit R, Wasiullah Malaviya D, Pandiyan K, Singh U, Sahu A, Shukla R, Singh B, Rai J, Sharma P, Lade H, Paul D (2015). Bioremediation of heavy metals from soil and aquatic environment: an overview of principles and criteria of fundamental processes. Sustainability.

[CR17] Drauz K, Gröger H, May O (2012). Enzyme catalysis in organic synthesis: a comprehensive handbook.

[CR18] Duran N, Marcato PD, Duran M, Yadav A, Gade A, Rai M (2011). Mechanistic aspects in the biogenic synthesis of extracellular metal nanoparticles by peptides, bacteria, fungi, and plants. Appl Microbiol Biotechnol.

[CR19] Erasmus M, Cason ED, Marwijk J, Botes E, Gericke M, Heerden E (2014). Gold nanoparticle synthesis using the thermophilic bacterium *Thermus scotoductus* SA-01 and the purification and characterization of its unusual gold reducing protein. Gold Bull.

[CR20] Eustis S, El-Sayed MA (2006). Why gold nanoparticles are more precious than pretty gold: noble metal surface plasmon resonance and its enhancement of the radiative and nonradiative properties of nanocrystals of different shapes. Chem Soc Rev.

[CR21] Faramarzi MA, Sadighi A (2013). Insights into biogenic and chemical production of inorganic nanomaterials and nanostructures. Adv Colloid Interface Sci.

[CR22] Farhan SN, Khadom AA (2015). Biosorption of heavy metals from aqueous solutions by *Saccharomyces cerevisiae*. Int J Ind Chem.

[CR23] Gadd GM (2010). Metals, minerals and microbes: geomicrobiology and bioremediation. Microbiology (Reading, England).

[CR24] Gangula A, Podila R, Karanam L, Janardhana C, Rao AM (2011). Catalytic reduction of 4-nitrophenol using biogenic gold and silver nanoparticles derived from *Breynia rhamnoides*. Langmuir.

[CR25] Gericke M, Pinches A (2006). Biological synthesis of metal nanoparticles. Hydrometallurgy.

[CR26] Gericke M, Pinches A (2006). Microbial production of gold nanoparticles. Gold Bull.

[CR27] Hulkoti NI, Taranath TC (2014). Biosynthesis of nanoparticles using microbes—a review. Colloids Surf B.

[CR28] Ivanov MR, Bednar HR, Haes AJ (2009). Investigations of the mechanism of gold nanoparticle stability and surface functionalization in capillary electrophoresis. ACS Nano.

[CR29] Jha AK, Prasad K, Kulkarni AR (2009). Synthesis of TiO_2_ nanoparticles using microorganisms. Colloids Surf B.

[CR30] Jha AK, Prasad K, Prasad K (2009). A green low-cost biosynthesis of Sb_2_O_3_ nanoparticles. Biochem Eng J.

[CR31] Ji X, Song X, Li J, Bai Y, Yang W, Peng X (2007). Size control of gold nanocrystals in citrate reduction: the third role of citrate. J Am Chem Soc.

[CR32] Kaliaperumal T (2011) PhD Thesis, Indian Institute of Technology Madras

[CR33] Kaliaperumal T, Kumar S, Gummadi SN, Chadha A (2010). Asymmetric synthesis of (*S*)-ethyl-4-chloro-3-hydroxybutanoate using *Candida parapsilosis* ATCC 7330. J Ind Microbiol Biotechnol.

[CR34] Kalimuthu K, Suresh Babu R, Venkataraman D, Bilal M, Gurunathan S (2008). Biosynthesis of silver nanocrystals by *Bacillus licheniformis*. Colloids Surf B.

[CR35] Kitching M, Ramani M, Marsili E (2014). Fungal biosynthesis of gold nanoparticles: mechanism and scale up. Microb Biotechnol.

[CR36] Kumar S, Gandhi KS, Kumar R (2007). Modeling of formation of gold nanoparticles by citrate method. Ind Eng Chem Res.

[CR37] Mahajabeen P, Chadha A (2013). A novel green route for the synthesis of *N*-phenylacetamides, benzimidazoles and acridinediones using *Candida parapsilosis* ATCC 7330. RSC Adv.

[CR38] Manivasagan P, Venkatesan J, Kang KH, Sivakumar K, Park SJ, Kim SK (2015). Production of *α*-amylase for the biosynthesis of gold nanoparticles using *Streptomyces* sp. MBRC-82. Int J Biol Macromol.

[CR39] Mittal AK, Kaler A, Mulay AV, Banerjee UC (2013). Synthesis of gold nanoparticles using whole cells of *Geotrichum candidum*. J Nanoparticles.

[CR40] Mukherjee P, Ahmad A, Mandal D, Senapati S, Sainkar SR, Khan MI, Ramani R, Parischa R, Ajayakumar PV, Alam M, Sastry M, Kumar R (2001). Bioreduction of AuCl_4_^−^ ions by the fungus, *Verticillium* sp. and surface trapping of the gold nanoparticles formed. Angew Chem Int.

[CR41] Nel AE, Madler L, Velegol D, Xia T, Hoek EMV, Somasundaran P, Klaessig F, Castranova V, Thompson M (2009). Understanding biophysicochemical interactions at the nano-bio interface. Nat Mater.

[CR42] Niide T, Goto M, Kamiya N (2011). Biocatalytic synthesis of gold nanoparticles with cofactor regeneration in recombinant *Escherichia coli* cells. Chem Commun.

[CR43] Panigrahi S, Basu S, Praharaj S, Pande S, Jana S, Pal A, Ghosh SK, Pal T (2007). Synthesis and size-selective catalysis by supported gold nanoparticles: study on heterogeneous and homogeneous catalytic process. J Phys Chem C.

[CR44] Penninckx MJ (2002). An overview on glutathione in *Saccharomyces* versus non-conventional yeasts. FEMS Yeast Res.

[CR45] Peters J, Zelinski T, Minuth T, Kula MR (1993). Synthetic applications of the carbonyl reductases isolated from *Candida parapsilosis* and *Rhodococcus erythropolis*. Tetrahedron Asymmetry.

[CR46] Pfeiffer C, Rehbock C, Hühn D, Carrillo-Carrion C, de Aberasturi DJ, Merk V, Barcikowski S, Parak WJ (2014). Interaction of colloidal nanoparticles with their local environment: the (ionic) nanoenvironment around nanoparticles is different from bulk and determines the physico-chemical properties of the nanoparticles. J R Soc Interface.

[CR47] Philip D (2009). Biosynthesis of Au, Ag and Au-Ag nanoparticles using edible mushroom extract. Spectrochim Acta Mol Biomol Spectrosc.

[CR48] Prathna TC, Chandrasekaran N, Mukherjee A (2011). Studies on aggregation behaviour of silver nanoparticles in aqueous matrices: effect of surface functionalization and matrix composition. Colloids Surf A.

[CR49] Rajeshkumar S, Malarkodi C, Gnanajobitha G, Paulkumar K, Vanaja M, Kannan C, Annadurai G (2013). Seaweed-mediated synthesis of gold nanoparticles using *Turbinaria conoides* and its characterization. J Nanostructure Chem.

[CR50] Reith F, Lengke MF, Falconer D, Craw D, Southam G (2007). The geomicrobiology of gold. ISME J.

[CR51] Riddin TL, Govender Y, Gericke M, Whiteley CG (2009). Two different hydrogenase enzymes from sulphate-reducing bacteria are responsible for the bioreductive mechanism of platinum into nanoparticles. Enzyme Microb Technol.

[CR52] Romero-Puertas MC, Corpas FJ, Sandalio LM, Leterrier M, Rodríguez-Serrano M, Del Río LA, Palma JM (2006). Glutathione reductase from pea leaves: response to abiotic stress and characterization of the peroxisomal isozyme. New Phytol.

[CR53] Roussos S, Soccol CR, Pandey A, Augur C (2013). New horizons in biotechnology.

[CR54] Scott D, Toney M, Muzikar M (2008). Harnessing the mechanism of glutathione reductase for synthesis of active site bound metallic nanoparticles and electrical connection to electrodes. J Am Chem Soc.

[CR55] Sen K, Sinha P, Lahiri S (2011). Time dependent formation of gold nanoparticles in yeast cells: a comparative study. Biochem Eng J.

[CR56] Sennett P, Olivier JP (1965). Colloidal dispersions, electrokinetic effects, and the concept of zeta potential. Ind Eng Chem.

[CR57] Seshadri S, Saranya K, Kowshik M (2011). Green synthesis of lead sulfide nanoparticles by the lead resistant marine yeast, *Rhodosporidium diobovatum*. Biotechnol Prog.

[CR58] Shamsaie A, Jonczyk M, Sturgis J, Paul Robinson J, Irudayaraj J (2007). Intracellularly grown gold nanoparticles as potential surface-enhanced Raman scattering probes. J Biomed Opt.

[CR59] Shankar SS, Ahmad A, Pasricha R, Sastry M (2003). Bioreduction of chloroaurate ions by geranium leaves and its endophytic fungus yields gold nanoparticles of different shapes. J Mater Chem.

[CR60] Sharma SK, Mudhoo A (2010). Green chemistry for environmental sustainability.

[CR61] Singh OV (2015). Bio-nanoparticles: biosynthesis and sustainable biotechnological implications.

[CR62] Singh R, Shedbalkar U, Wadhwani S, Chopade B (2015). Bacteriagenic silver nanoparticles: synthesis, mechanism, and applications. App Microbiol Biotechnol.

[CR64] Tan YN, Lee JY, Wang DIC (2010). Uncovering the design rules for peptide synthesis of metal nanoparticles. J Am Chem Soc.

[CR63] Vaidyanathan R, Gopalram S, Kalishwaralal K, Deepak V, Pandian SR, Gurunathan S (2010). Enhanced silver nanoparticle synthesis by optimization of nitrate reductase activity. Colloids Surf B.

[CR65] Venkataraman S, Chadha A (2015). Biocatalytic deracemisation of aliphatic beta-hydroxy esters: improving the enantioselectivity by optimisation of reaction parameters. J Ind Microbiol Biotechnol.

[CR66] Vetchinkina EP, Loshchinina EA, Burov AM, Dykman LA, Nikitina VE (2014). Enzymatic formation of gold nanoparticles by submerged culture of the basidiomycete *Lentinus edodes*. J Biotechnol.

[CR67] Virkutyte J, Varma RS (2013) Green synthesis of nanomaterials: environmental aspects sustainable nanotechnology and the environment: advances and achievements. In: ACS symposium series, vol 1124. American Chemical Society, p 11–39

[CR68] Wang J, Chen C (2009). Biosorbents for heavy metals removal and their future. Biotechnol Adv.

[CR69] Wang J, Zhang G, Li Q, Jiang H, Liu C, Amatore C, Wang X (2013). In vivo self-bio-imaging of tumors through in situ biosynthesized fluorescent gold nanoclusters. Sci Rep.

[CR70] Weast RC (1984). CRC handbook of chemistry and physics.

[CR71] Yadav A, Kon K, Kratosova G, Duran N, Ingle AP, Rai M (2015). Fungi as an efficient mycosystem for the synthesis of metal nanoparticles: progress and key aspects of research. Biotechnol Lett.

[CR72] Yang S, Wang Y, Wang Q, Zhang R, Ding B (2007). UV irradiation induced formation of Au nanoparticles at room temperature: the case of pH values. Colloids Surf A.

[CR73] Zayed MF, Eisa WH (2014). *Phoenix dactylifera* L. leaf extract phytosynthesized gold nanoparticles; controlled synthesis and catalytic activity. Spectrochim Acta Mol Biomol Spectrosc.

