# Supplementary material for: Whole resting cells vs. cell free extracts of Candida parapsilosis ATCC 7330 for the synthesis of gold nanoparticles
Source: AMB Express. 2016 Oct 7;6:92. doi: 10.1186/s13568-016-0268-y (PMC5055519; doi:10.1186/s13568-016-0268-y)
Supplement: Supplementary file 1 — Additional file 1. Additional figures and tables. [file 13568_2016_268_MOESM1_ESM.pdf]

AMB Express

# Whole resting cells *vs.* Cell free extracts of *Candida parapsilosis* ATCC 7330 for the synthesis of gold nanoparticles

Saravanan Krishnan[1], Shoba Narayan[1, 2] and Anju Chadha [1, 3, 4 *]

*Correspondence: Anju Chadha
Phone: 91-44-2257 4106; Fax: 91-44-2257 4102
E. mail: anjuc@iitm.ac.in

Saravanan Krishnan
E. mail: sara.krish87@gmail.com

Shoba Narayan
E. mail: shobulu@gmail.com

[1] Laboratory of Bioorganic Chemistry, Department of Biotechnology, Indian Institute of Technology Madras, Chennai, India

[2] Present Address: Faculty of Allied Health Sciences, Chettinad Academy of Research and Education, Kelambakkam, Chennai, India

[3] National Center for Catalysis Research, Indian Institute of Technology Madras, Chennai, India

[4] Centre for NEMS and Nanophotonics, Indian Institute of Technology Madras, Chennai, India

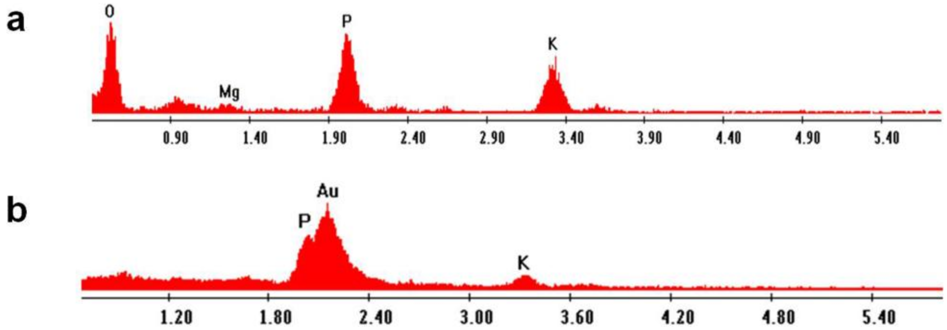


**Fig. S1** Energy dispersive analysis of the yeast biomass (a) Control cells and (b) cells after the reaction with gold (III) chloride

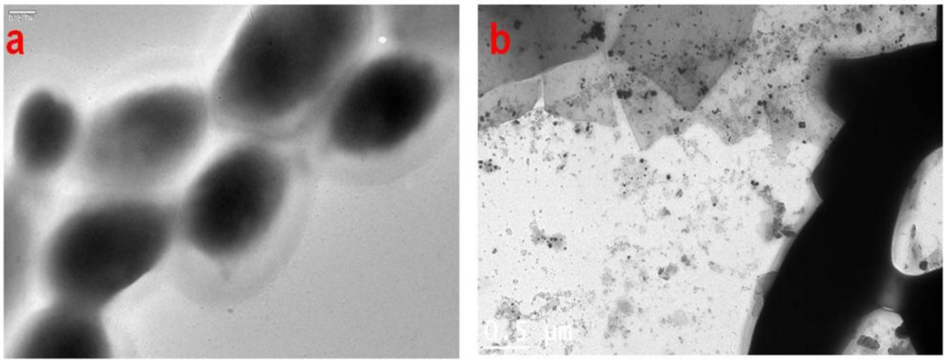


**Fig. S2** Transmission electron micrographs (a) heat killed cells of *Candida parapsilosis* ATCC 7330 before and (b) heat killed cells after the reaction (right) with gold (III) chloride

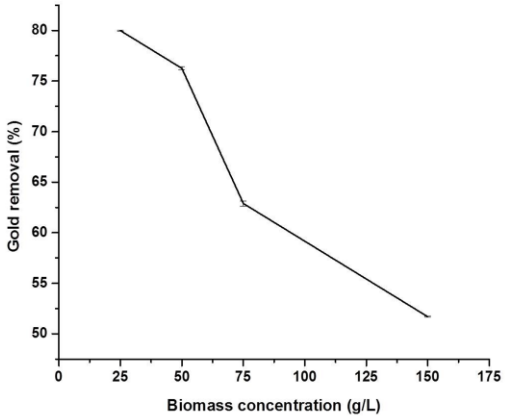


**Fig. S3** Effect of biomass concentration of *Candida parapsilosis* ATCC 7330 on the uptake of gold (III) ions

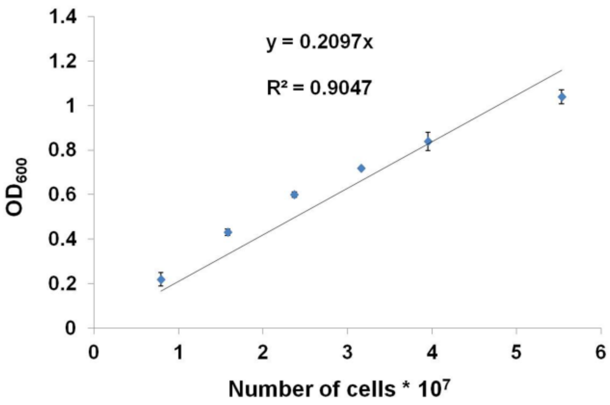


**Fig. S4** Calibration of the optical density measurements to the number of yeast cells

**Table S1.** Estimation of the number of gold atoms accumulated in a single *Cp* cell

| Entry | Number of cells (x $10^9$) [A] | Average gold uptake (%) | Gold atoms uptake by cell (x $10^{20}$) [B] | Average uptake in single cell* (x $10^{10}$) [B]/[A] |
|---|---|---|---|---|
| 1 | 1.8 | 29.44 | 1.033 ± 0.045 | 5.74 |
| 2 | 3.6 | 39.29 | 1.38 ± 0.007 | 3.83 |
| 3 | 5.4 | 43.37 | 1.52 ± 0.014 | 2.81 |
| 4 | 7.2 | 50.09 | 1.76 ± 0.013 | 2.44 |
| 5 | 9 | 51.13 | 1.79 ± 0.028 | 1.99 |
| 6 | 10.8 | 51.97 | 1.825 ± 0.007 | 1.69 |

**Table S2.** Extraction of the biosynthesized gold nanoparticles formed within the whole cells using ultrasonication

| Method | Procedure | Follow-up |
|---|---|---|
| Ultrasonication | Ultrasonication for 20 mins (1 sec On/Off) 35 % amplitude | When centrifuged at low speed (~500 g), the nanoparticles was not observed in the supernatant fraction<br>Final step: Filtration<br>Identical samples filtered through Whatman filter and 0.2 μm syringe filter. The filtrate was colourless (no nanoparticles released). |

**Powder X-ray diffraction analysis**

The above figure represents the diffraction peaks noticed at Bragg's angle of the gold nanoparticles as 111, 200, 220, 311, 222 lattice planes which manifests the face centered cubic (FCC) nature of the gold nanocrystals obtained.

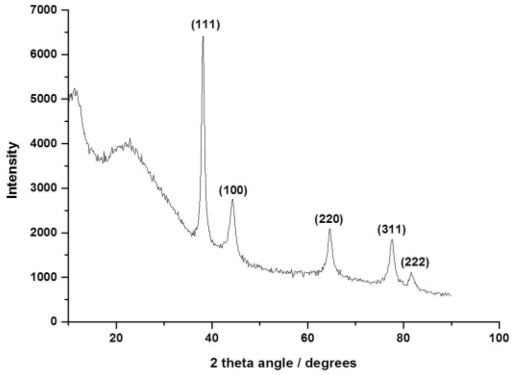


**Fig. S5** Powder X-Ray diffraction of the gold nanoparticles biosynthesized

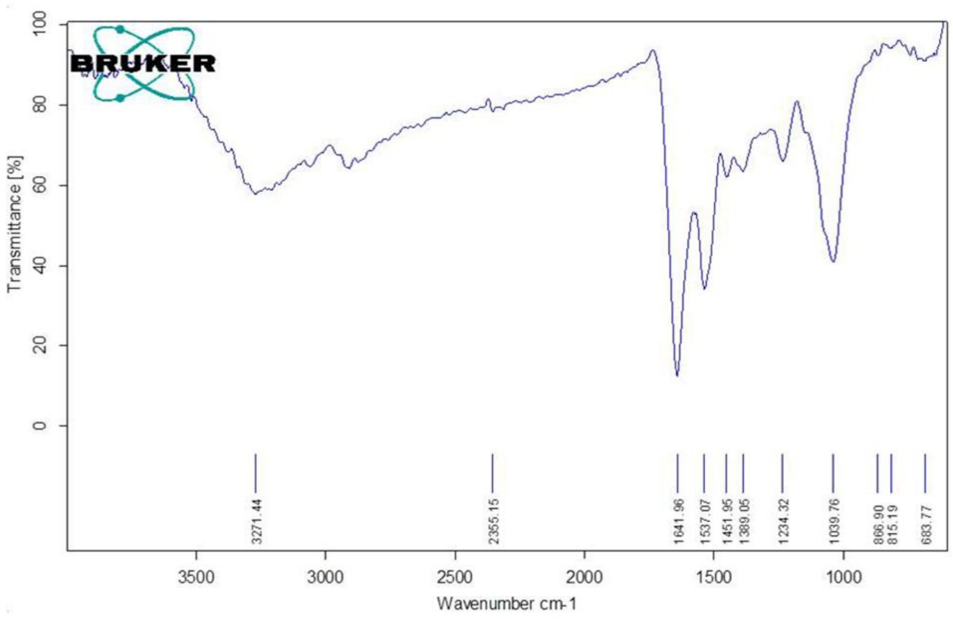


**Fig. S6** Fourier Transform - Infra Red Spectroscopy analysis of the biosynthesized gold nanoparticles

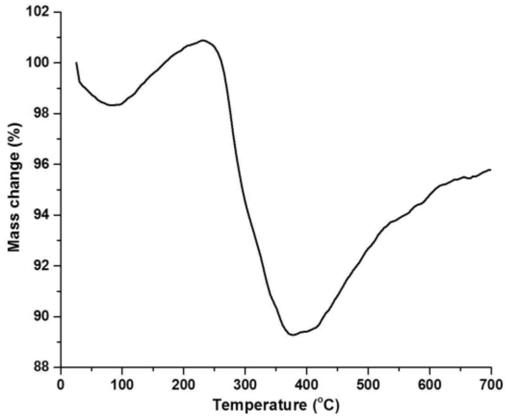


**Fig. S7** Thermal gravimetric analysis of the lyophilized sample of bio-synthesized gold nanoparticles

**SDS-PAGE analysis (12%)**

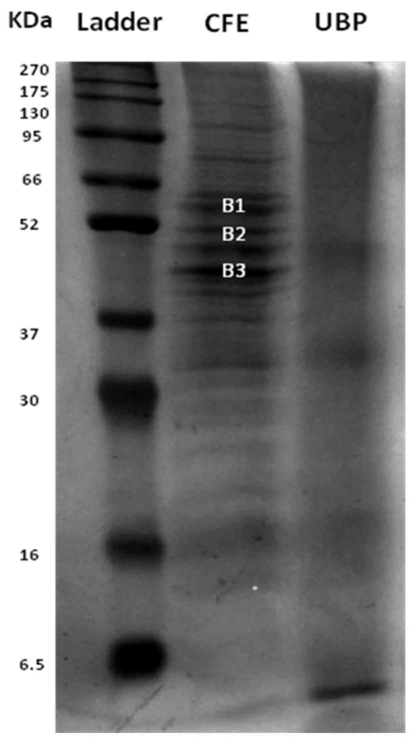


**Fig. S8** SDS-PAGE (12%) analysis of the cell free extracts (CFE) and unbound proteins (UBP)

| Entry | Average relative intensity of protein in UBP* |
|---|---|
| B1 | 0.13 |
| B2 | 0.09 |
| B3 | 0.15 |

* Cell free extract is the reference sample

**Table.S3** Relative intensity of proteins in the unbound proteins fraction as compared to the cell free extract based on densitometry analysis using Image lab 3.0 software

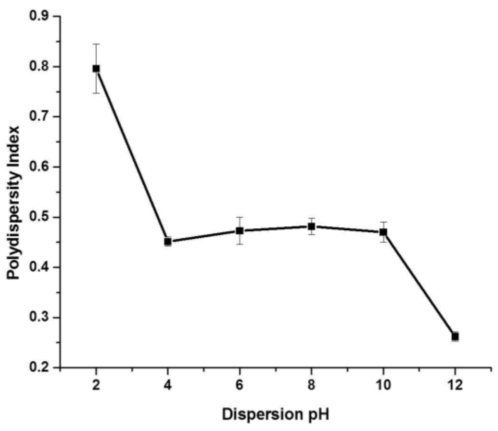


**Fig. S9** Plot of polydispersity index as a function of pH of the dispersion medium

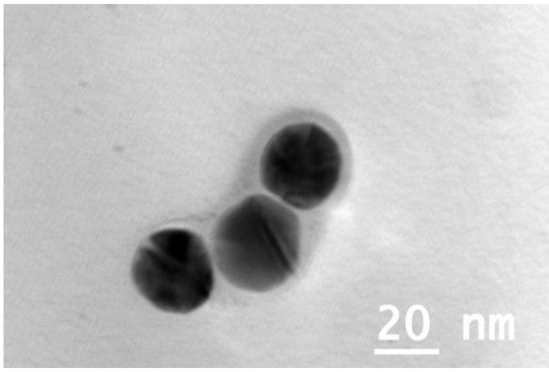


**Fig. S10** Electron micrographs of the pH 12 dispersed gold nanoparticles after 20 months storage (Scale bar: 20nm, Magnification: 150Kx)

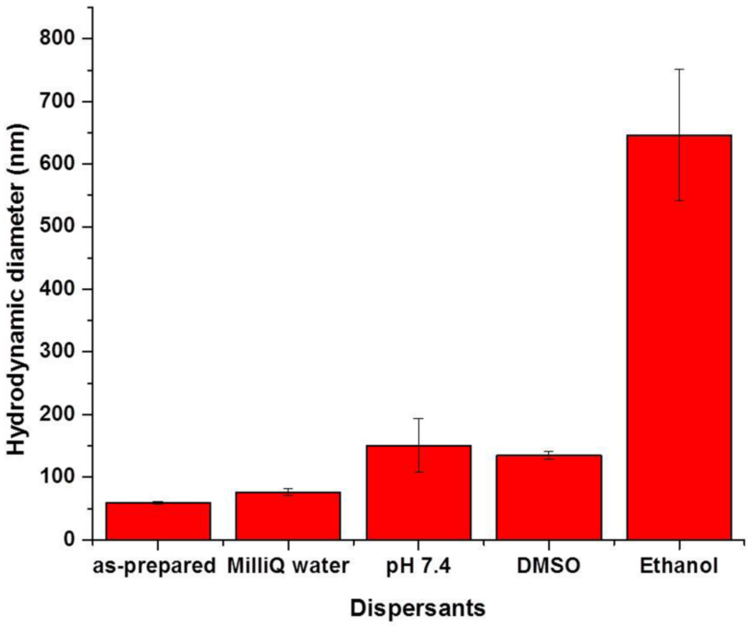


**Fig. S11** Hydrodynamic diameter of the biosynthesized gold nanoparticles dispersed in various media

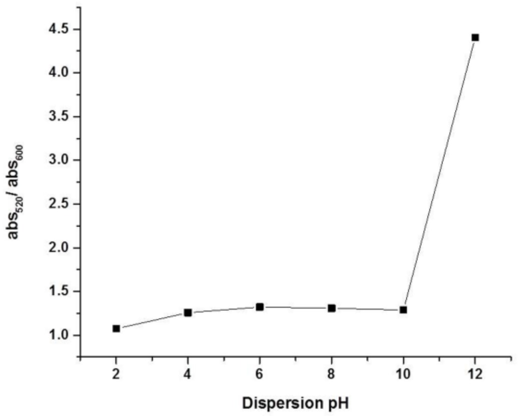


**Fig. S12** Plot of stability parameter *vs* pH to examine the dispersion stability of the gold nanoparticles in different pH solutions

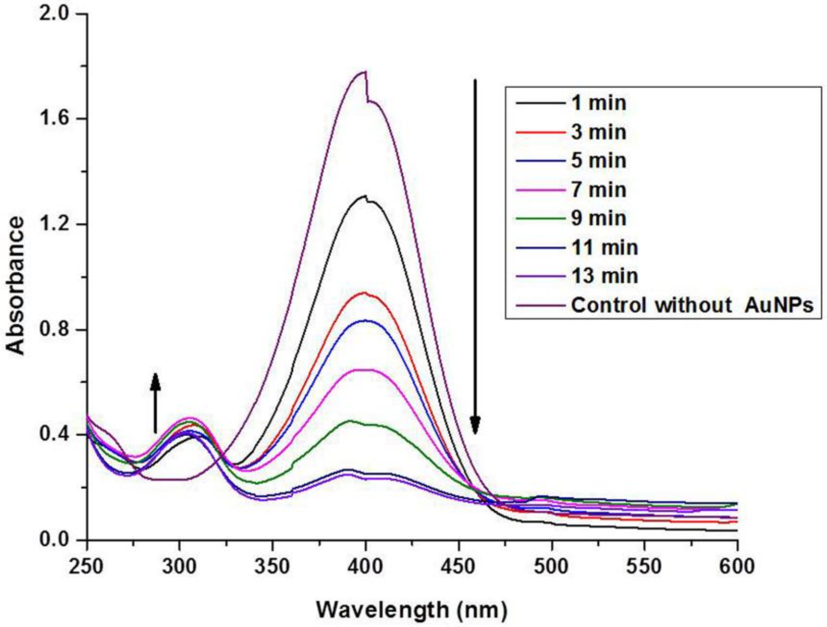


**Fig. S13** Spectrophotometric monitoring of the sodium borohydride mediated reduction of 4-nitrophenol to 4-aminophenol using gold nanoparticles as a catalyst

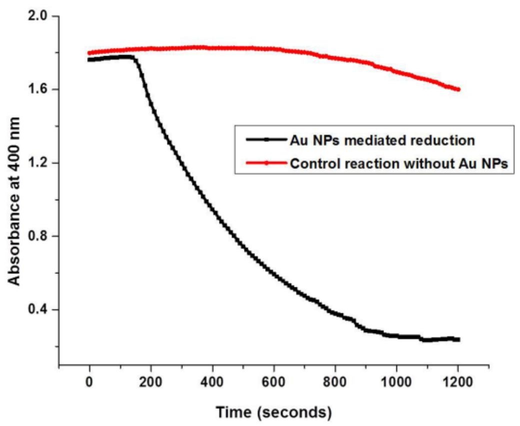


**Fig. S14** Time course measurements of the catalytic reduction of 4-nitrophenol recorded at 400 nm
